# The unique N-terminal region of *Mycobacterium tuberculosis* sigma factor A plays a dominant role in the essential function of this protein

**DOI:** 10.1016/j.jbc.2023.102933

**Published:** 2023-01-20

**Authors:** Biplab Singha, Debashree Behera, Mehak Zahoor Khan, Nitesh Kumar Singh, Divya Tej Sowpati, Balasubramanian Gopal, Vinay Kumar Nandicoori

**Affiliations:** 1 National Institute of Immunology, New Delhi, India; 2 CSIR-Centre for Cellular and Molecular Biology, Hyderabad, India; 3 Molecular Biophysics Unit, Indian Institute of Science, Bangalore, India

**Keywords:** Transcription, Regulation, Sigma factor, Mycobacteria, Tuberculosis, SigA, SigB, AES, allelic exchange substrate, ATc, anhydrotetracycline, CFU, colony forming unit, DEG, differentially expressed gene, Dox, doxycycline, ECF, extra cytoplasmic σ factor, IVN, isovaleronitrile, Mtb, Mycobacterium tuberculosis, p.i, post infection, PCA, principal component analysis, RNAP, RNA polymerase, SPR, surface plasmon resonance, WCL, whole-cell lysate

## Abstract

SigA (σ^A^) is an essential protein and the primary sigma factor in *Mycobacterium tuberculosis* (*Mtb*). However, due to the absence of genetic tools, our understanding of the role and regulation of σ^A^ activity and its molecular attributes that help modulate *Mtb* survival is scant. Here, we generated a conditional gene replacement of σ^A^ in *Mtb* and showed that its depletion results in a severe survival defect *in vitro*, *ex vivo*, and *in vivo* in a murine infection model. Our RNA-seq analysis suggests that σ^A^ either directly or indirectly regulates ∼57% of the *Mtb* transcriptome, including ∼28% of essential genes. Surprisingly, we note that despite having ∼64% similarity with σ^A^, overexpression of the primary-like σ factor SigB (σ^B^) fails to compensate for the absence of σ^A^, suggesting minimal functional redundancy. RNA-seq analysis of the *Mtb* σ^B^ deletion mutant revealed that 433 genes are regulated by σ^B^, of which 283 overlap with the σ^A^ transcriptome. Additionally, surface plasmon resonance, *in vitro* transcription, and functional complementation experiments reveal that σ^A^ residues between 132-179 that are disordered and missing from all experimentally determined σ^A^-RNAP structural models are imperative for σ^A^ function. Moreover, phosphorylation of σ^A^ in the intrinsically disordered N-terminal region plays a regulatory role in modulating its activity. Collectively, these observations and analysis provide a rationale for the centrality of σ^A^ for the survival and pathogenicity of this *bacillus*.

*Mycobacterium tuberculosis (Mtb)* enters the body through nasal airways and deposits in the lower lungs, wherein the primarily alveolar macrophages engulf the bacteria (1). In response to the host's dynamic microenvironment, *Mtb* remodels its gene expression to facilitate its survival within the host. Gaining insights into how *Mtb* modulates its transcriptional machinery to survive under hostile host conditions is imperative for tackling this deadly pathogen. Gene expression is primarily regulated at the transcription initiation step in bacteria. Transcription initiation involves many diverse molecular interactions that allow the apo RNA polymerase (RNAP) to recognize the promoter and facilitate DNA unwinding around the transcription start-point. Contrary to eukaryotes, wherein three RNAP are present, bacteria encode only one RNAP consisting of 2 subunits of α, one subunit each of β, β′, and a ω subunit (2). Even though core RNAP is sufficient for transcriptional elongation, it cannot initiate transcription without a σ factor (3). σ factors play a crucial role in promoter recognition and initiating the melting process of promoter regions (4, 5, 6, 7).

*Mtb* encodes for one essential and twelve nonessential σ factors, classified into four groups based on their domain architecture (8, 9) (Fig. S1). Group 4 mainly comprises ECF (extra cytoplasmic σ factor) σ^C^ to σ^M^ except for σ^F^. σ^F^ and σ^B^ are the sole members of groups 3 and 2, respectively (9, 10, 11). Essential σ factor, σ^A^, belong to group 1, containing four α-helical domains. In addition, it harbors a long N-terminal extension (Fig. S1). Studies have previously shown that N-terminal (1.1) region in *Escherichia coli* σ^70^ (RpoD) inhibits its binding to promoters in the absence of RNAP (12). However, there is little sequence similarity between RpoD and *Mycobacterium smegmatis* σ^A^ (σ^A^_Msm_), especially in the N-terminal region. Furthermore, the N-terminal region of σ^A^_Msm_ is predicted to be intrinsically disordered (13). The overexpression of *sigA* enhances bacterial survivability inside macrophages and mice lungs. The expression of anti-sense *sigA* reduces the bacillary load (14).

The highly conserved amino acid residues in the subdomains 2.4 and 4.2 of σ^A^ directly interact with −10 and −35 regions of the promoter (13). In the case of ECF σ factors, the cognate anti-σ factors sequester these initiation factors, thus regulating their activity (15). However, the regulation of σ^A^ has not been investigated yet. Besides σ factors, mycobacterial transcription is modulated by transcription factors such as RbpA, CarD, and WhiB proteins, specific to actinomycetes (16, 17, 18, 19, 20, 21). These transcription factors interact with σ^A^ to regulate transcription, and disrupting these interactions slow down the transcription process (16, 21, 22). The presence of RbpA increases the affinity of σ^A^ for RNAP caused by the interaction of σ^A^ and RbpA. Hubin *et al.* predicted the interacting amino acid residues of σ^A^_Msm_ from the structure of the RbpA-σ^A^_Msm_–DNA complex (13). It was also shown that interaction between σ^A^ and DevR enables bacteria to survive hypoxia (23). The C-terminal domain of σ^A^ interacts with various transcriptional factors such as WhiB. Furthermore, σ^A^-mediated activation of the *eis* gene (Rv2416) results in enhanced intracellular growth of the W-beijing strain of mycobacteria (24).

While the σ^A^ expression is constitutive, the expression of σ^B^ is upregulated during stationary conditions (25, 26). However, during exponential growth conditions, σ^B^ expression levels are comparable with σ^A^ (27). σ^A^ and σ^B^ share significant sequence similarity (∼64%) identity between the structured DNA recognition regions of σ^A^ and σ^B^, including −10 and −35 region-binding amino acid residues. Deletion of *sigB* renders the pathogen sensitive to heat, oxidative, surface stress, and antibiotics (28). However, it does not impact the survival of *Mtb* in macrophages or mouse lungs (1, 9, 26, 28). Chip Seq data suggested that several promoters are shared by σ^A^ and σ^B^ (27). σ^A^ and σ^B^ are both phosphorylated (29, 30); however, the importance of this modification in regulating their activity remains elusive.

Collectively, our knowledge regarding the structure-function and regulatory aspects of σ^A^ in *Mtb* is poor. Here, we set out to answer a few critical questions related to the functionality of σ^A^ in *Mtb* and its functional correlation with σ^B^. We sought to answer the following questions: (i) What is the effect of depleting σ^A^ on the survival of *Mtb* within the host? (ii) What is the effect of σ^A^ depletion and σ^B^ KO on the global transcriptome of *Mtb* and functional redundancy of σ^A^ and σ^B^?, (iii) Can overexpression of σ^B^ rescue phenotypic defects observed upon σ^A^ depletion?, (iv) The role of the unique N-terminal extension of σ^A^? (v) How does phosphorylation modulate σ^A^ function? Here, we describe results from experiments designed to address these aspects of σ^A^-mediated modulation of the expression profile in *Mtb*. A highlight is the intriguing finding that a disordered extended N-terminal polypeptide, hitherto ‘unseen’ in structural descriptions of the *Mtb* RNAP complex, plays a dominant role in σ^A^ activity.

## Results

### σ^A^ is essential for Mtb survival

**Figure 1:**
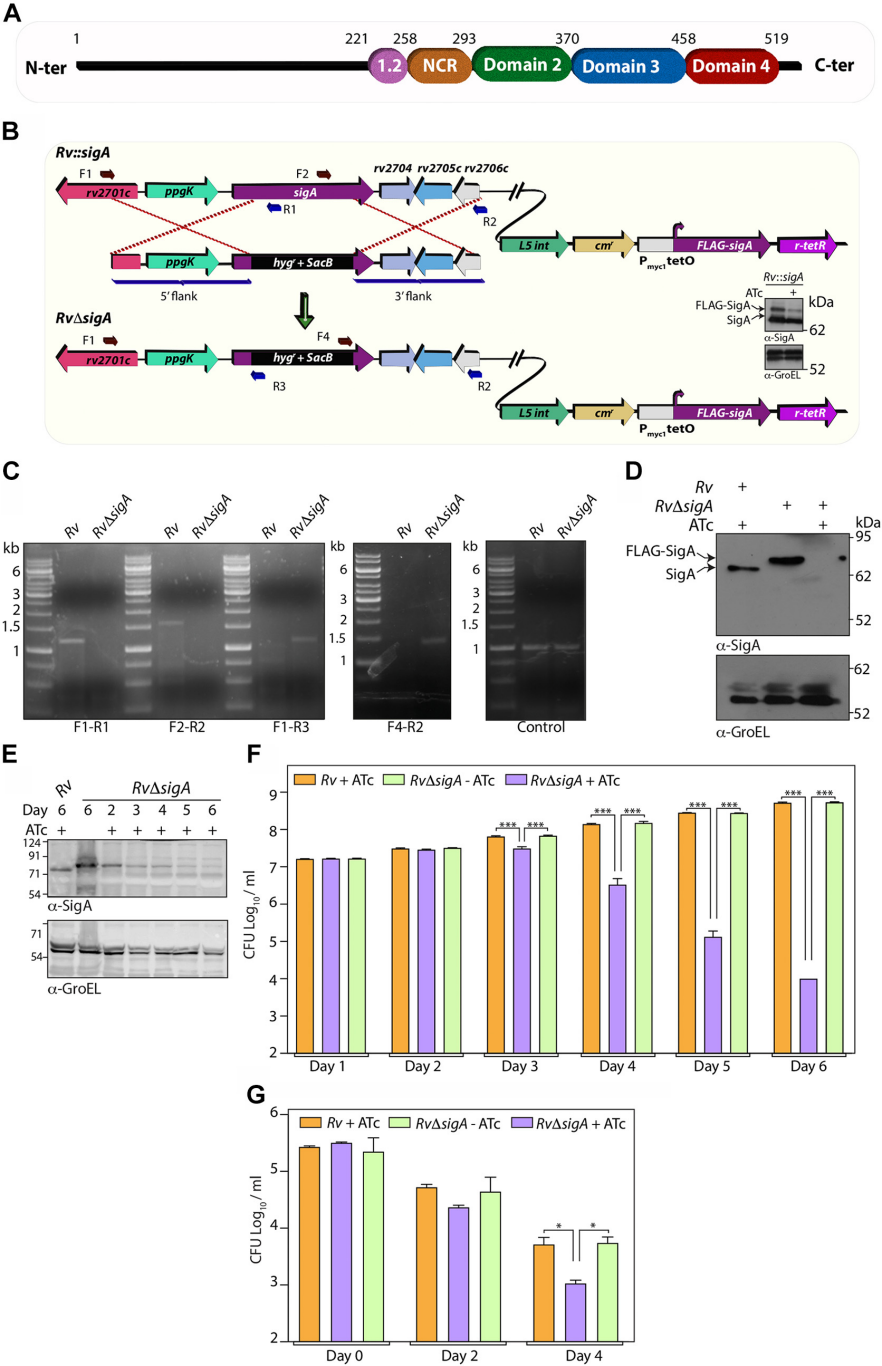
**σ**^**A**^**is essential for Mtb survival.***A*, schematic overview of the domain architecture of *Mtb* SigA. *B*, schematic depicting homologous recombination between the Allelic Exchange Substrate (AES) and *sigA* native genomic loci (*rv2703*). Legitimate recombination was confirmed by performing multiple PCRs. Primer used for the confirmation are depicted. Inlet shows the immunoblot, wherein *Rv::sigA* merodiploid was grown in the absence or presence of ATc. Whole-cell lysates (WCLs) were resolved on 10% SDS-PAGE, transferred to nitrocellulose membrane, and probed with α-SigA (10,000-fold dilution)^61^ and α-GroEL2 (10,000-fold dilution) antibodies generated in house. The band corresponding to endogenous and FLAG-tagged SigA are indicated by *arrows*. *C*, agarose gel images depicting PCR amplification from genomic DNA isolated from *Rv* or *RvΔsigA* with multiple indicated primer sets. Amplification with F1-R1 or F2-R2 primers results in 1.2 kb or 1.6 kb respectively from *Rv* but none with *RvΔsigA*, and amplification with F1-R3 or F4-R2 primers results in 1.1 kb or 1.4 kb respectively from *RvΔsigA* but none with *Rv. D*, 30 μg of WCLs from *Rv* or *RvΔsigA* were resolved on 10% SDS-PAGE, transferred to the membrane, and probed with α-sigA and α-GroEL antibodies. *E*, *Rv* or *RvΔsigA* grown in the absence of ATc were seeded at A_600_ ∼0.05, and the cultures were grown in the absence or presence of ATc. WCLs were prepared second day onward post ATc addition. The WCLs were resolved and probed with α-SigA and α-GroEL antibodies. *F*, *Rv* or *RvΔsigA* cultures were seeded at A_600_ ∼0.05 in 7H9-ADC, and the growth *in vitro* was monitored by enumerating CFU every day for 6 days. The data represent mean CFU log_10_/ml ± SD of three independent replicates. ∗∗∗, *p* < 0.0001. *G*, peritoneal macrophage were isolated from BALB/c mice and infected with *Rv* or *RvΔsigA* at 1:5 MOI. The cells were washed with RPMI 4 h post infection (p.i) and RPMI with or without ATc. The intracellular bacillary load was enumerated on Day 0, 2, and 4 p.i. Data information: The data represent mean CFU log_10_/ml ± SD of three independent replicates. Statistical significance was drawn in comparison with *RvΔsigA* using one-way ANOVA (Tukey test; GraphPad prism 9). ∗, *p* < 0.01; ∗∗∗, *p* < 0.0001. ATc, anhydrotetracycline; CFU, colony forming unit; Mtb, *Mycobacterium tuberculosis*.

σ^A^ is a 528 amino acid essential protein with an extended 207 amino acid–long disordered N terminal region preceding the four α helical domains (Fig. 1*A*). Domain 2 interacts with the −10-promoter element, domain 3 with the extended −10 promoter region, and domain 4 interacts with the −35 region (Fig. S1). The function of domain 1, consisting of nonconserved regions and the extended N-terminal region, has not yet been elucidated (Figs. 1*A* and S1). To investigate structure-function relationships and the *in vitro* and *in vivo* role of σ^A^, we sought to generate a conditional gene replacement mutant (*RvΔsigA*). *sigA* was cloned into pFICTO, and the construct was electroporated into *Mtb-H37Rv* (*Rv*) to generate merodiploid strain (*Rv::sigA*) (Fig. 1*B*). The expression of FLAG-σ^A^ from integrative copy is under anhydrotetracycline (ATc) regulation. In the absence of ATc, the P_smyc_ promoter (31) is active, and upon the addition of ATc, the r-TetR–ATc complex binds to the Tet operator sequences in the promoter, shutting down transcription. Western Blot analysis confirmed that the expression of FLAG-σ^A^ from the merodiploid is significantly lowered upon the addition of ATc (Fig. 1*B*). Subsequently, *sigA* at the native loci was replaced with a *hyg*^*r*^*-sacB* cassette with the help of a specialized transduction methodology to generate *RvΔsigA* (32). The fidelity of recombination at the native loci was confirmed by PCR and Western blot analysis (Fig. 1, *C* and *D*). Analysis of FLAG-σ^A^ levels at different days post-ATc addition revealed a significant reduction from day 3 (Fig. 1*E*). *In vitro* growth analysis suggested that while the growth of *RvΔsigA* was comparable to *Rv* in the absence of ATc, depletion of FLAG-σ^A^ resulted in compromised growth, eventually resulting in a 4-log fold (10,000-fold) difference between the growth of parental and mutant strain (Fig. 1*F*). Importantly, depletion of FLAG-σ^A^ (*RvΔsigA* +ATc) significantly reduced the intracellular survival in the peritoneal macrophages as compared to either *Rv* or *RvΔsigA*-ATc–infected cells (Fig. 1*G*). Together, these results confirm the generation of conditional *RvΔsigA* mutant and the criticality of σ^A^ for both *in vitro* and *ex vivo* survival of the pathogen.

### σ^A^ is essential for Mtb survival *in vivo*

**Figure 2:**
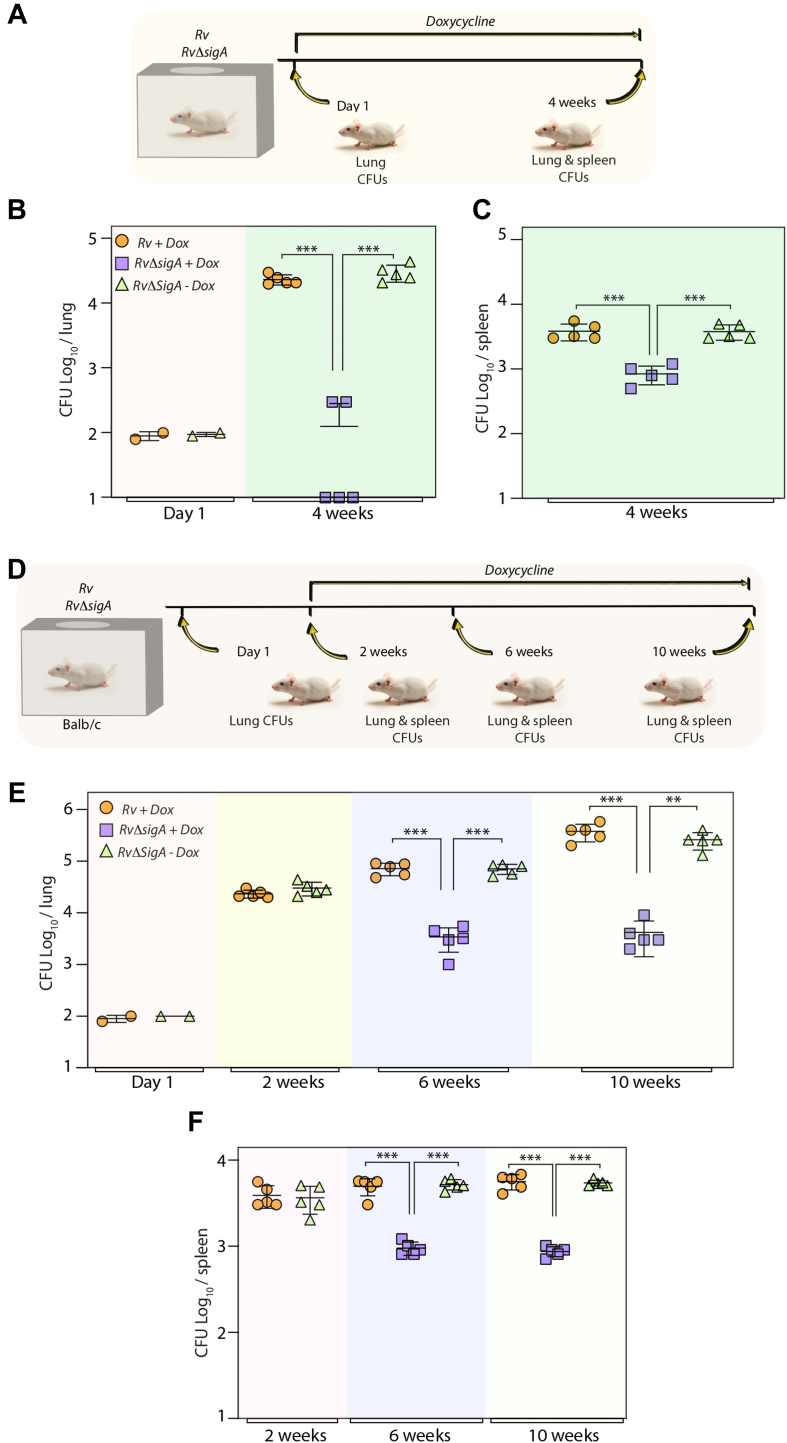
**σ**^**A**^**is essential for Mtb survival *in vivo*.***A*, schematic depicting the outline of *in vivo* murine infection. Doxycycline (Dox) was introduced in the water on 1-day p.i. *B* and *C*, BALB/c mice were aerosolically challenged with 100 CFU/mice (10^8^ bacteria) of *Rv* or *RvΔsigA*, and bacillary survival was enumerated in the lung (*B*) & spleen (*C*) homogenates at day 1 and 4 weeks p.i. Each data point indicates CFU log_10_/lung or spleen obtained from one mice, and error bar represents as mean CFU log_10_/ml ± SD. ∗, *p* < 0.01; ∗∗, *p* < 0.001; ∗∗∗, *p* < 0.0001. *D*, schematic depicting the outline of experiment. Dox was introduced after establishment of the infection, *i.e.*, at day 14. *E* and *F*, BALB/c mice were infected with 100 CFU/mice of *Rv* or *RvΔsigA*, and the infection was established for 14 days. Dox was administrated to *Rv* and one set of *RvΔsigA* (n = 5) infected mice, while other set was *left* untreated. CFU was enumerated on day 1, 2-, 6-, and 10-weeks p.i in the lung (*E*) and spleen (*F*) homogenates. Data information: Each data point indicates CFU log_10_/lung or spleen obtained from one mice, and error bar represents the mean CFU log_10_/lung or spleen ± SD. Statistical significance was drawn in comparison with *RvΔsigA* using one-way ANOVA (Tukey test; GraphPad prism 9). ∗∗, *p* < 0.001; ∗∗∗, *p* < 0.0001. CFU, colony forming unit; Mtb, *Mycobacterium tuberculosis*.

We used a murine infection model to evaluate *in vivo* survival of *the RvΔsigA* mutant. Mice infected with *Rv or RvΔsigA* through the aerosolic route and the colony forming units (CFUs) enumerated 24 h post infection (p.i) indicated efficient and equivalent implantation of WT and mutant bacilli in the lungs of mice (Fig. 2, *A* and *B*). To examine the impact of depleting σ^A^, a set of animals infected with *RvΔsigA* were provided doxycycline (Dox) in the drinking water (Fig. 2*A*). The data suggest that depletion of σ^A^ from the start of infection results in a significant reduction (2.5–3.5 log_10_ fold) in the bacillary load (*RvΔsigA* + Dox) at 4 weeks p. i compared with either *Rv* + Dox or *RvΔsigA* - Dox in both lungs and spleen of the infected animals (Fig. 2, *B* and *C*). We sought to examine the impact of depleting σ^A^ from an established infection. Mice were infected with *Rv or RvΔsigA*, and the infection was allowed to be established for 2 weeks (Fig. 2*D*). Subsequently, *RvΔsigA*-infected animals were divided into four groups; wherein two groups were given Dox in the water for the subsequent weeks, and the CFUs were enumerated (Fig. 2*D*). Compared with *Rv* + Dox *or RvΔsigA* -Dox, a decrease of 1.5 and 2.5 log_10_ fold was observed in the lungs of *RvΔsigA* +Dox mice at 6- and 10-weeks p. i, respectively (Fig. 2*E*). A similar trend was observed in the mice spleens (Fig. 2*F*). Together, this data suggests that while σ^A^ is critical for the survival of *Mtb* within the host, the impact of depleting σ^A^ is less significant during the chronic stage of infection.

### σ^A^ depletion impacts global transcription in Mtb

**Figure 3:**
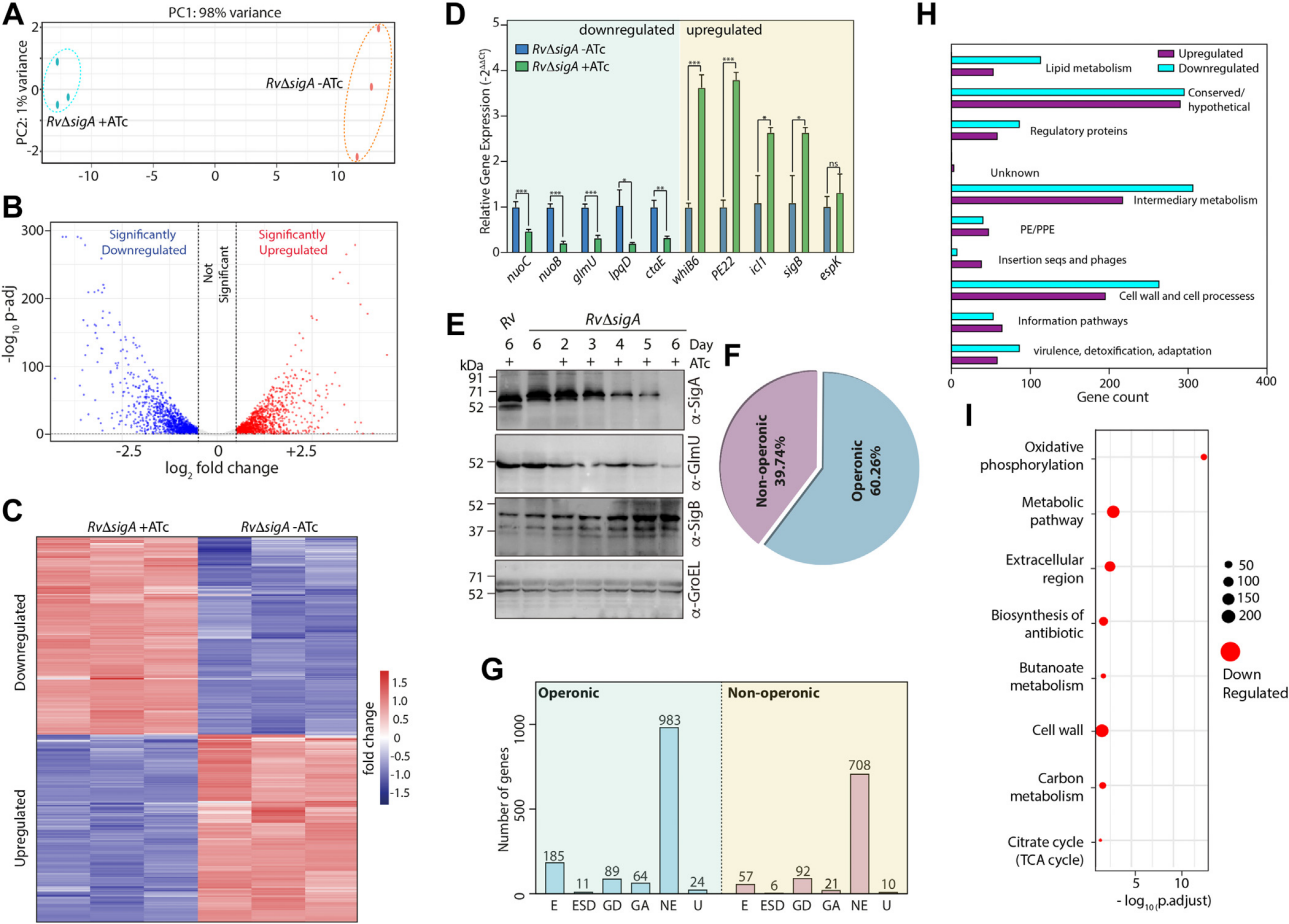
**σ**^**A**^**depletion impacts global transcription in Mtb.***A*, principal component analysis (PCA) of transcriptomics data sets from two distinct group of samples (*RvΔsigA* – ATc and *RvΔsigA* + ATc). *B*, volcano plot representing differentially expressed genes (DEGs) in *RvΔsigA* +ATc compared with *RvΔsigA*-ATc. *Red* spots indicate the genes that are upregulated and *blue* spots indicated those are downregulated (padj < 0.05, log_2_ fold change = 0.5). *C*, heat maps showing the normalized read counts of DEGs from three biological replicates of *Rv* and *RvΔsigA.* Color intensity indicates relative upregulation (*blue*) or downregulation (*red*). *D*, five each upregulated and downregulated genes from RNA-seq analysis were validated with the help of qRT-PCR. Data information: data were normalized with respect to 16s rRNA and is plotted as mean ± SD (performed in triplicates (n = 3)). Statistical significance was analyzed using Student's *t* test (two-tailed, unpaired). ∗, *p* < 0.01; ∗∗, *p* < 0.001; ∗∗∗, *p* < 0.0001. *E*, *Rv* and *RvΔsigA* WCLs were prepared on different days post ATc from cultures grown for 6 days in 7H9 in the presence or absence of 1 μg/ml ATc. WCLs from *Rv, RvΔsigA* were resolved and probed with α-SigA, α-SigB, and α-glmU. *F*, the pie chart depicting the percentage of total DEGs that are operonic and nonoperonic. *G*, bar graph showing the number of operonic and nonoperonic DEGs that belong to essential (E), essential domain (ED), growth defect (GD), growth advantage (GA), non-essential (NE), and uncertain (U) categories. *H*, bar graphs depicting the numbers of upregulated (*purple*) and downregulated (*blue*) DEGs plotted that belong to a various functional categories. *I*, gene enrichment analysis shows the number of genes in various biological pathways that are upregulated or downregulated upon depletion of SigA (FDR < 0.05). ATc, anhydrotetracycline; Mtb, *Mycobacterium tuberculosis*; WCL, whole-cell lysate.

σ^A^ is proposed to regulate majority of the mycobacterial transcriptome under normal growth conditions. To identify the regulons and pathways that σ^A^ modulates, we performed RNA-seq of *RvΔsigA* grown *in vitro* in the absence and presence of ATc. Towards this, RNA was extracted from *RvΔsigA* cultured in the absence or presence of ATc for 3 or 4 days. The RNA obtained 4 days post-ATc addition was degraded, and hence we used RNA extracted from *RvΔsigA* in the absence or presence of ATc on day 3 for the experiment. The experiment was performed in biological triplicates, and the principal component analysis (PCA) showed appropriate clustering of *RvΔsigA*-ATc and *RvΔsigA* +ATc samples (Fig. 3*A*). The data analysis showed that *sigA* expression was downregulated ∼0.7 fold in *RvΔsigA* +ATc samples (padj = 2.79E-08). To identify differentially expressed genes (DEGs), we applied a cut-off as padj <0.05 and absolute log_2_FC >0.5. There were 2320 DEGs, among which 1133 genes were upregulated, and 1187 genes were downregulated upon depletion of σ^A^ (Fig. 3*B* and Table S1). Heat maps for each biological *RvΔsigA* − ATc and *RvΔsigA* + ATc sample show a complete set of upregulated and downregulated genes (Fig. 3*C*). To validate the RNA-seq analysis results, we selected five upregulated and downregulated genes each and performed a qRT-PCR analysis with *RvΔsigA* − ATc and *RvΔsigA* + ATc samples (Fig. 3*D*). To further validate this observation, Western blot analysis of σ^B^ and GlmU, which are upregulated and downregulated, respectively, in *RvΔsigA* on different days post-ATc addition were examined (Fig. 3*E*). These results were consistent with RNA sequence data, wherein we observed an increase in the protein levels of σ^B^ and a decrease in the levels of glmU at the later time points post-ATc addition (Fig. 3*E*).

Next, we assessed the operonic arrangement of the 2320 protein coding DEGs and found that 39.74% of the total were nonoperonic, while 60.26% were operonic (Fig. 3*F*). Of the 242 essential genes differentially regulated upon σ^A^ depletion, 57 were nonoperonic and 185 operonic (Fig. 3*G*). Pathway analysis suggested that many DEGs belong to multiple pathways, including lipid metabolism, intermediary metabolism, and cell wall and cell processes (Fig. 3*H*). Gene enrichment analysis indicated that critical metabolic pathways such as oxidative phosphorylation (ETC), TCA cycle, nucleic acid biosynthesis, and cell wall synthesis pathways are substantially enriched upon depletion in downregulated genes (Fig. 3*I* and Table S2). Together, it appears that σ^A^ regulates the expression of ∼1/3rd of the essential genes in *Mtb*. More importantly, it regulates the expression of many genes involved in cell wall synthesis, oxidative phosphorylation, and TCA cycles. Thus, results suggest that σ^A^ either directly or indirectly regulates ∼57% of *Mtb* genes and that encompasses most critical cellular processes. It is thus not surprising that its depletion results in cell death.

### σ^B^ overexpression fails to rescue the σ^A^ depletion phenotype

**Figure 4:**
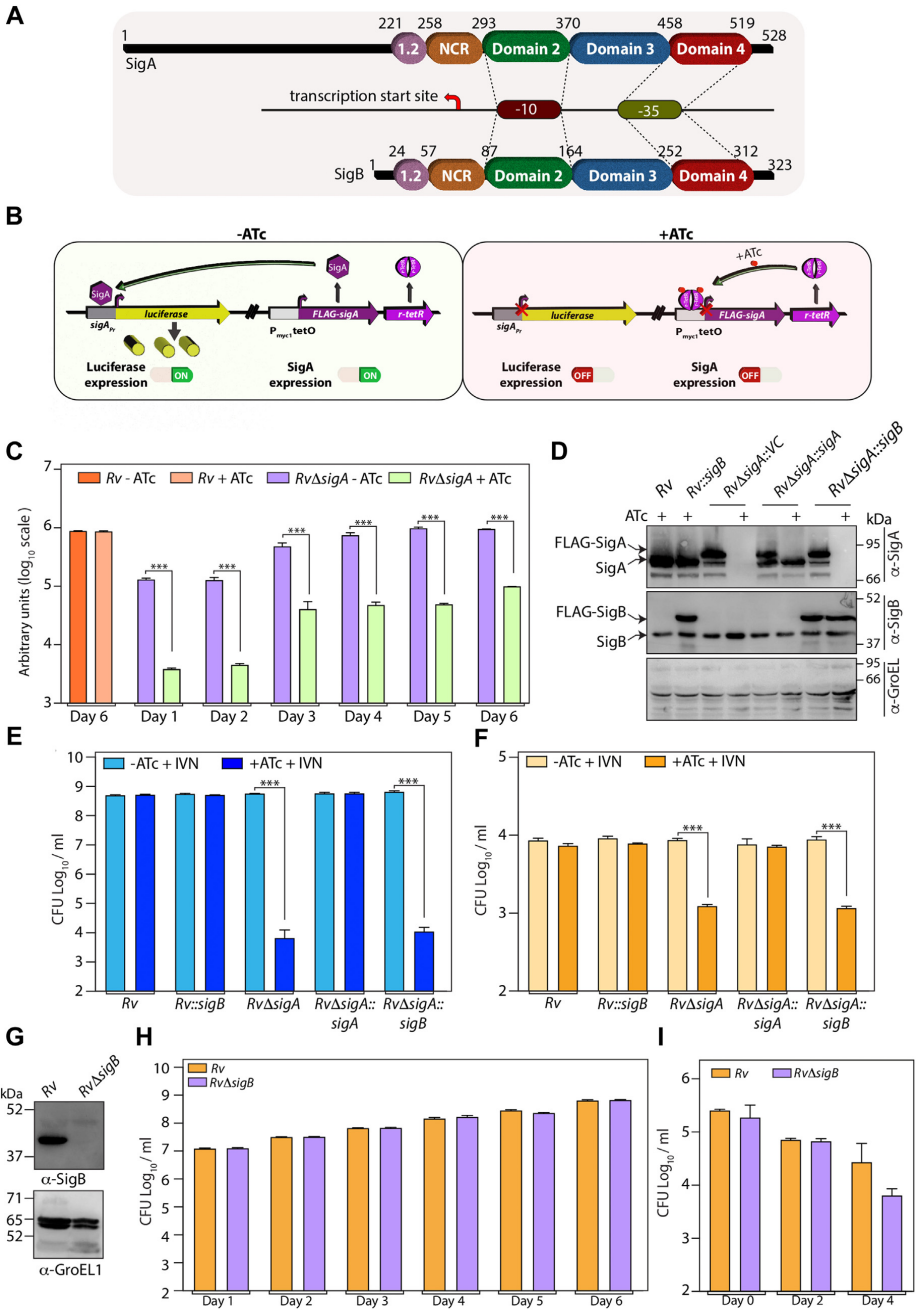
***sigB* overexpression fails to rescue *sigA* depletion phenotype.***A*, schematic showing the overlap between various domains of SigA and SigB. *B*, the promoter of *sigA* was fused to luciferase gene in pSW vector. *Rv* and *RvΔsigA* were electroporated with pSW-luc_SApr_ to generate *Rv::luc*_*SApr*_ and *RvΔsigA::luc*_*SApr*_ strains. *C*, luciferase activity was measured from WCLs prepared from cultures on different days post ATc addition. The experiment was performed in triplicates. Data were normalized with respect to 16s rRNA and is plotted as mean ± SD (performed in triplicates (n = 3).Statistical significance was analyzed using Student's *t* test (two-tailed, unpaired). ∗∗∗, *p* < 0.0001. *D*, *Rv* and *RvΔsigA* strains were electroporated with pNiT-3XF and pNiT-3XF-sigB to generate *Rv::F-sigB* and *RvΔsigA::F-sigB*. WCLs were prepared from cultures grown for 6 days in 7H9 + 0.2 μM IVN, in the presence or absence of 1 μg/ml ATc. WCLs from *Rv, RvΔsigA*, and *RvΔsigA*_wt/mut_ were resolved and probed. *E*, *Rv, Rv::sigB, RvΔsigA*, and *RvΔSigA::F-sigB* cultures grown in 7H9-ADC + 0.2 μM IVN, in the absence or presence of ATc for 6 days and CFUs were enumerated. The data represent mean CFU log_10_/ml ± SD of three independent replicates. *F*, murine peritoneal macrophage cells were infected with *Rv, Rv::sigB, RvΔsigA*, and *RvΔSigA::F-sigB* at 1:5 MOI. 0.2 μM IVN was added to induce the expression of episomally produced SigA, and ATc was added wherein indicated to repress FLAG-SigA expression. CFUs were enumerated at 96 h post infection. The data represent mean CFU log_10_/ml ± SD of three biological replicates (n = 3). *G*, 30 μg of WCLs from *Rv* and *RvΔsigB* were resolved and probed with α-sigB and α-GroEL antibodies. *H*, *Rv* and *RvΔsigB* cultures were inoculated at 0.05 A in 7H9 + 0.2 μM IVN, in the absence or presence of 1.0 μg/ml ATc, and CFUs were enumerated on day 6. The data represent mean CFU log_10_/ml ± SD of three independent replicates. *I*, murine peritoneal macrophage cells were infected with *Rv, Rv::sigB, RvΔsigA*, and *RvΔSigA::sigB* at 1:5 MOI. Post wash, cells were replenished with RPMI + 0.2 μM IVN, ± 1.0 μg/ml ATc, and CFUs were enumerated at 96 h p.i. Data information: the data represent mean CFU log_10_/ml ± SD of three independent replicates. Statistical significance analyzed using one-way ANOVA (Tukey test; GraphPad prism 9). ∗∗∗, *p* < 0.0001. ATc, anhydrotetracycline; CFU, colony forming unit; IVN, isovaleronitrile; WCL, whole-cell lysate.

RNA-seq analysis and Western blot analysis (Fig. 3) suggested that depletion of σ^A^ led to an increase in the expression levels of σ^B^. σ^B^ is the only group II σ factor in *Mtb*, with ∼64% identity with σ^A^. The significant difference is the absence of the N-terminal extended region in σ^B^ (Fig. 4*A*). To ascertain whether the higher levels of σ^B^ influence the expression of proteins from σ^A^-regulated promoters, we performed a reporter assay. The upstream promoter region up to the start codon of *sigA* (500 bp) was fused with the ORF of luciferase in an integrative shuttle vector. The construct was electroporated into *Rv* and *RvΔsigA* (Fig. 4*B*). The luciferase activity in the lysates was evaluated on different days post ATc in addition to evaluating the impact of σ^A^ depletion on the expression of σ^A^-modulated promoter. There was a significant reduction in the luciferase activity (compared with -ATc samples) on days 1 and 2 post σ^A^ depletion. However, on the subsequent days, the activity was higher (Fig. 4*C*). We reasoned that higher expression of σ^B^ is likely responsible for the increased promoter activity upon σ^A^ depletion. This hypothesis aligns with a previous report, which suggested a significant overlap in the promoter utilization between σ^A^ and σ^B^ (27). If this is indeed true, overexpression of σ^B^ should compensate for the absence of σ^A^. σ^B^ overexpression construct (pNit-F-SigB) was transformed into *Rv* and *RvΔsigA*, and the expression of FLAG-σ^B^ was confirmed by Western blotting analysis (Fig. 4*D*). The overexpression of σ^B^ failed to mitigate the phenotypic defects observed upon depletion of σ^A^ both *in vitro* and *ex vivo* (Fig. 4, *E* and *F*), suggesting a unique regulatory function for σ^A^. Thus, we set out to examine the extent of uniqueness/redundancy of these σ factors. To address this question, we generated a mutant of *sigB, RvΔsigB,* wherein *the sigB* gene was replaced with a *hyg*^*r*^ antibiotic marker (Fig. S2*A*). The replacement of sigB at the native loci was confirmed by multiple PCRs (Fig. S2*B*) and Western blot (Fig. 4*G*). *In vitro* growth (Fig. 4*H*) and *ex vivo* infection (Fig. 4*I*) experiments suggested that the presence of σ^B^ was dispensable for *Mtb* survival. Together, data suggest that the function of σ^A^ is distinctive, and the absence of σ^B^ has no particular impact on growth.

### σ^B^-regulated transcriptome only partially overlaps with *σ*^A^

**Figure 5:**
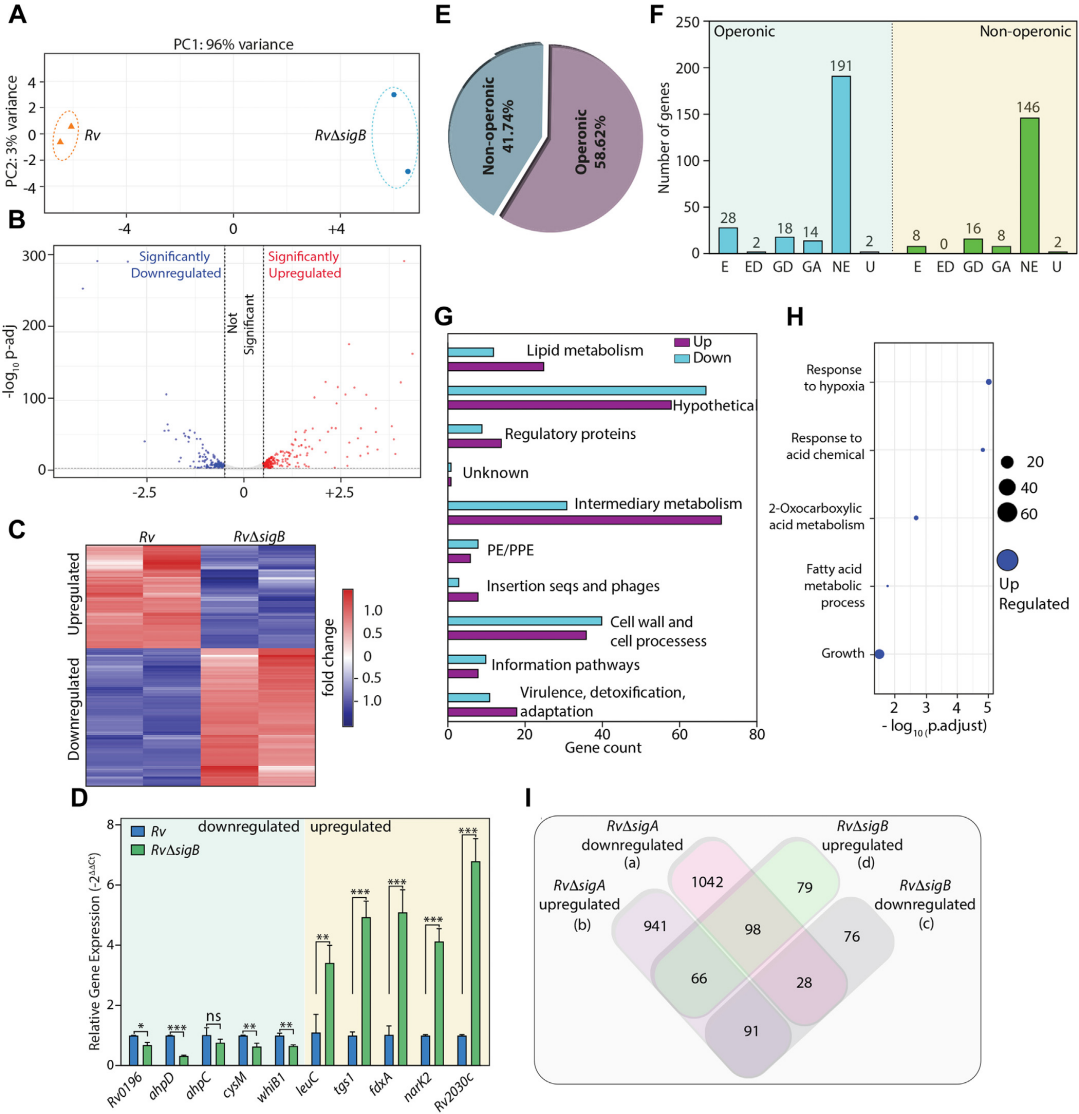
**sigB depletion impacts global transcription in Mtb.***A*, principal component analysis (PCA) of transcriptomics data sets from two distinct group of samples (*Rv* and *RvΔsigB*). *B*, volcano plot representing differentially expressed genes (DEGs) in *Rv* compared with *RvΔsigB*. *Red* spots indicate the genes that are upregulated and *blue* spots indicates genes that are downregulated (padj < 0.05, log_2_ fold change (FC) = 0.5). *C*, heat maps showing the normalized read counts of DEGs from two biological replicates of *Rv and RvΔsigB.* Color intensity indicates relative upregulation or downregulation, where *blue* indicated upregulated genes and *red* indicate downregulated genes. *D*, five selected upregulated and downregulated from RNA-seq analysis were validated with the help of qRT-PCR. Data were normalized with respect to 16s rRNA and is plotted as mean ± SD (performed in triplicates (n = 3)). Statistical significance was analyzed using Student's *t* test (two-tailed, unpaired). ∗, *p* < 0.01; ∗∗, *p* < 0.001; ∗∗∗, *p* < 0.0001. *E*, pie chart showing the percentage of DEGs that are operonic and nonoperonic. *F*, bar graph showing the number of operonic and nonoperonic DEGs that belong to essential (E), essential domain (ED), growth defect (GD), growth advantage (GA), nonessential (NE), and uncertain (U) categories. *G*, bar graphs depicting the numbers of unregulated (*purple*) and downregulated (*blue*) DEGs that belong to a different functional category. *H*, gene enrichment analysis of DEGs revealed upregulation of five biological pathways in the absence of *sigB* (FDR < 0.05). *I*, Venn diagram showing the overlapping protein-coding genes regulated by *sigA* and *sigB*. Conditions in Venn diagram are (*A*) genes downregulated upon *sigA* depletion, (*B*) genes upregulated upon *sigA* depletion, (*C*) genes downregulated in the absence of *sigB*, and (*D*) genes upregulated in the absence of *sigB.* Mtb, *Mycobacterium tuberculosis*.

Next, we sought to examine the commonality in downstream gene expression between σ^A^ and σ^B^. RNA-seq analysis was performed in biological duplicates with the samples extracted from the log phase cultures of *Rv* and *RvΔsigB.* PCA analysis showed suitable clustering of *Rv* and *RvΔsigB* samples (Fig. 5*A*).Volcano plot represent 433 DEGs using a cut-off of padj < 0.05 and absolute log_2_FC > 0.5, among which 247 genes were upregulated and the 187 genes were downregulated in *RvΔsigB* (Fig. 5*B* and Table S3). The heat map shows that this trend is maintained for both the biological replicates of *Rv* or *RvΔsigB* (Fig. 5*C*). To validate the data, we performed qRT-PCR analysis of the top five upregulated and downregulated genes. The data obtained is in agreement with RNA-seq trends, even though the data for one sample was statistically insignificant (Fig. 5*D*). Analysis suggested that ∼61.9% of genes belong to operons, indicating plausible regulation of their promoters by σ^B^ (Fig. 5*E*).

Interestingly, among the 417 protein-coding DEGs, only 54 are essential genes (Fig. 5*F*). While we find genes corresponding to all the cellular processes among DEGs, hypothetical, intermediatory metabolism, and cell wall/synthesis process contribute the most (Fig. 5*G*). Gene enrichment analysis indicated enrichment of five different processes, including growth, only in upregulated genes (Fig. 5*H* and Table S4). Unlike σ^A^, the absence of σ^B^ did not show enrichment of any cellular processes in downregulated DEGs. Next, we overlapped the DEGs obtained upon *sigA* depletion with those obtained without *sigB.* It appears 98 and 28 genes are upregulated or downregulated in the absence of either σ factor. The data in Figure 4 shows upregulation of σ^B^ upon σ^A^ depletion, which may have contributed to upregulated DEGs. Those genes which σ^B^ regulates in the absence of σ^A^ are expected to overlap with downregulated DEGs in the absence of σ^B^. We observed 91 such genes in the overlap analysis (Fig. 5*I* and Table S5). Importantly, ∼2000 DEGs obtained upon depletion of σ^A^ and ∼150 DEGs obtained in the absence of σ^B^ were unique. The data thus suggest that σ^A^ and σ^B^ regulate many nonoverlapping genes.

### The N-terminal unstructured region is critical for σ^A^ functionality

**Figure 6:**
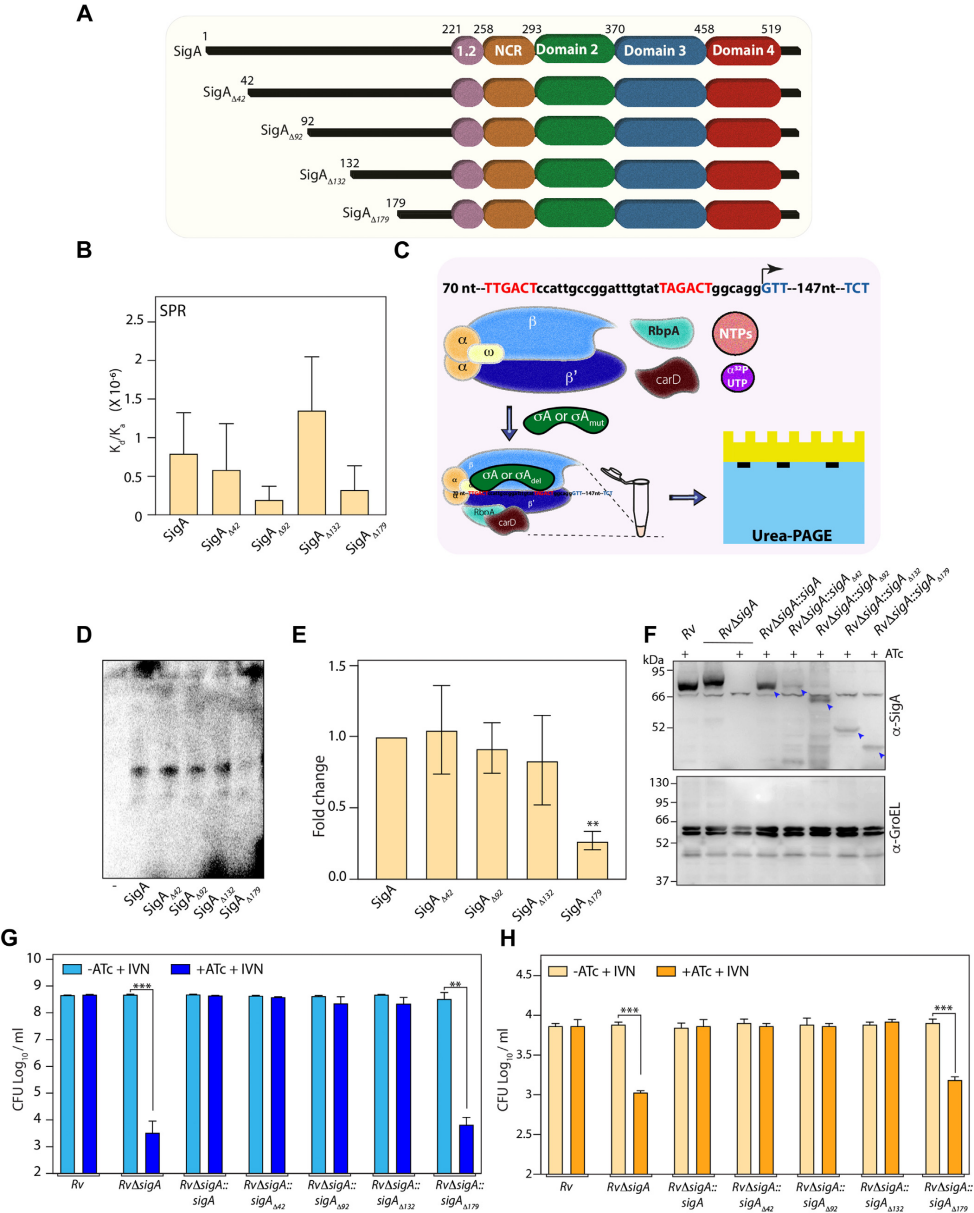
**The N-terminal unstructured region is critical for σ**^**A**^**function.***A*, schematic representation of full-length SigA and various N-terminal truncation mutants. *B*, binding affinity of various SigA mutants with *rrnAP3* promoter DNA derived from the SPR sensorgrams in Figure S4. *C*, schematic representation of *in vitro* transcription assay. 250 nt template DNA-harboring rrnAP3 promoter was mixed with holo RNA polymerase, RbpA, CarD, NTPs, α[^32^P] UTP, and SigA/SigA_Mut_. *D*, *in vitro* transcription assay performed in the presence or absence of either SigA or SigA_Mut_ were resolved on UREA-PAGE. Representative image of 150 nt–radiolabeled RNA product captured using phosphorimager. *E*, quantification of three biological replicates. *F*, *RvΔsigA* strain was electroporated with pNit-sigA, pNit-sigA_Δ42_, pNit-sigA_Δ92_, pNit-sigA_Δ132_, and pNit-sigA_Δ179_ to generate *RvΔsigA::sigA*, *RvΔsigA::sigA*_*Δ42*_, *RvΔsigA::sigA*_*Δ92*_, *RvΔsigA::sigA*_*Δ132*_, and *RvΔsigA::sigA*_*Δ179*_. WCLs were prepared from cultures inoculated at A ∼0.05 and grown for 6 days in the IVN and presence or absence of ATc. 30 μg of WCLs from *Rv, RvΔsigA*, and *RvΔsigA*_*wt/mut*_ were resolved and probed with α-sigA and α-GroEL antibodies. *G*, *Rv, RvΔsigA*, *and RvΔsigA*_*wt/mut*_ cultures inoculated A ∼0.05 in 7H9-ADC + 0.2 mM IVN, in the absence or presence of 1 μg/ml ATc, and CFUs were enumerated on day 6. The data represent mean CFU log_10_/ml ± SD of three independent replicates. *H*, murine peritoneal macrophage cells were infected with *Rv, RvΔsigA*, and *RvΔsigA*_*wt/mut*_ at 1:5 MOI. 4 h p.i, cells were washed and replenished with RPMI + 0.2 μM IVN media with or without 1 μg/ml ATc, and CFUs were enumerated at 96 h p.i. Data information: the data represent mean CFU log_10_/ml ± SD of three independent replicates. Statistical significance was analyzed using one-way ANOVA (Tukey test; GraphPad prism 9). *p* < 0.01; ∗∗, *p* < 0.001; ∗∗∗, *p* < 0.0001. ATc, anhydrotetracycline; CFU, colony forming unit; IVN, isovaleronitrile; SPR, surface plasmon resonance; WCL, whole-cell lysate.

The data presented in Figure 4, Figure 5 suggest that σ^B^ cannot complement σ^A^ despite having considerable homology in the four structured promoter recognition domains. As stated earlier, σ^A^ has a long 225 aa and unique N-terminal extension (33). This led us to examine the role of the N-terminal region in modulating the function of σ^A^. We generated a series of N-terminal truncation mutants, wherein we deleted either 42, 92, 132, or 179 aa from the N-terminus. The mutants were cloned into pNit1 and pET vectors to generate pNiT-*sigA*_*Δ*42_, pNiT-*sigA*_*Δ*92_, pNiT-*sigA*_*Δ*132_, and pNiT-*sigA*_*Δ*179_ and the corresponding pET vector constructs (Fig. 6*A*). RpbA, CarD, RNAP, σ^A^, and σ^A^ deletion mutants were expressed and purified from *E. coli.* CD studies suggested the absence of gross changes in secondary structure among the truncated σ^A^ variants (Fig. S3). σ^A^ is known to bind with the *rrnAP3* promoter element (13), and hence, biotinylated-AP3 promoter immobilized on streptavidin (SA) chip was used for determining the binding efficiency using Surface Plasmon Resonance (SPR). We first evaluated the ability of σ^A^ and σ^A^ deletion mutants to interact with the promoter region with the help of SPR. We did not observe statistically significant differences in the binding affinity between WT and deletion mutants of σ^A^ (Figs. 6*B* and S4). Next, we examined the impact of σ^A^ truncation mutants on their ability to initiate transcription. RNAP holoenzyme, RbpA, and CarD were purified, and *in vitro* transcription assay was performed in the presence or absence of σ^A^_wt/mut_. As expected, in the absence of σ^A^, the RNAP holoenzyme, RbpA, and CarD could not be recruited to the promoter and hence no product formation was observed (Fig. 6, *C*–*E*). Except for σ^A^_Δ179_, σ^A^ and the other σ^A^ deletion mutants could initiate transcription, suggesting that the residues between 132 and 179 are critical for σ^A^’s ability to initiate the transcription (Fig. 6, *D* and *E*). We hypothesized that residues between 132-179 are primarily acidic and may be involved in forming the transcription bubble necessary for transcription initiation. Subsequently, we examined the ability of σ^A^ deletion mutants to complement the function of σ^A^ *in vitro* and *ex vivo*. pNiT constructs were electroporated into *RvΔsigA,* and the expression of σ^A^ or σ^A^ N-terminal deletion constructs was evaluated by Western blot (Fig. 6*F*). While all the σ^A^ deletion mutants were seen to be expressed, their expression was lower than the full length (Fig. 6*F*). In line with our *in vitro* transcription data, all the mutants other than σ^A^_Δ179_ complemented the function of σ^A^ in the presence of ATc (Fig. 6*G*). The results obtained in *ex vivo* infection experiments were found to be similar to *in vitro* growth data (Fig. 6*H*). These results suggest that acidic amino acids between amino acids 132-179 residues play an important role in modulating the activity of σ^A^.

### Phosphorylation impacts σ^A^ function

**Figure 7:**
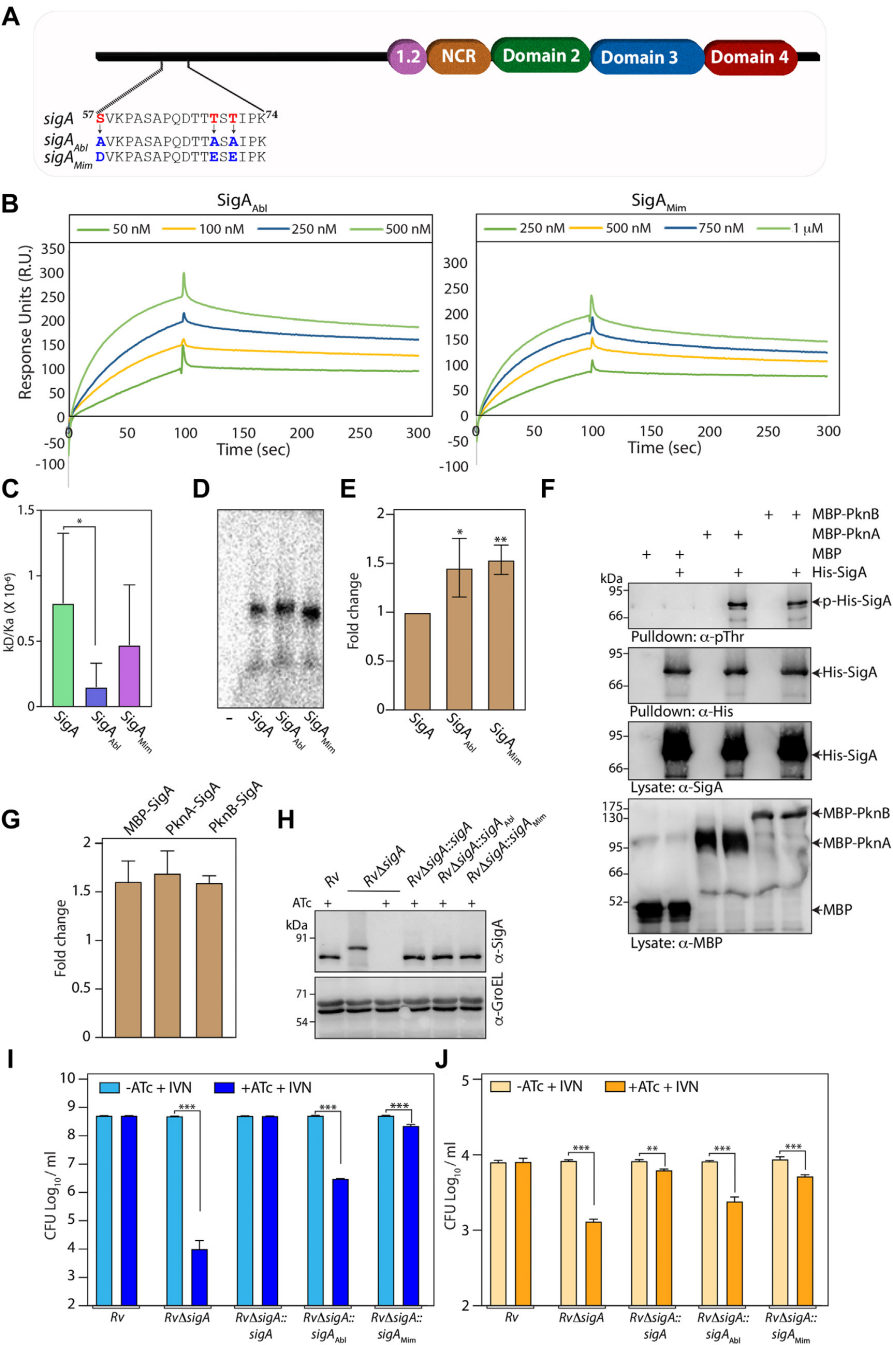
**Phosphorylation impacts σ**^**A**^**function.***A*, line diagram depicting the phosphorylation sites (marked in *red*) in the N-terminal region of of SigA. S57, T69, and T71 were mutated to A57, A69, and A71 to generate σ^A^ ablative mutant (σ^A^_*Abl*_) and to D57, E69, and E71, to generate σ^A^ mimetic mutant (σ^A^_*Mim*_). *B*, the SPR sensorgram of *rrnAP3* promoter DNA and σ^A^/SigA_Mut_ interactions. *C*, binding affinity of various SigA mutants with *rrnAP3* promoter DNA derived from the SPR sensorgrams in (*B*). SPR experiments using σ^A^-truncated variants and phosphomutants were performed in quadruplets at the same time. WT σ^A^ σ^A^-rrnAP3 promoter interaction control is the same for both sets (Figs. 6*B* and 7C). *D*, *in vitro* transcriptions assay as described above. The radiolabeled 150 nt mRNA product was resolved on Urea-PAGE and visualized by phosphor scanner and quantified using Quantity One. Figure provided is representative of one of three replicates. *E*, quantification of three biological replicate data from (*D*). *F*, pDuet constructs with MBP, MBP-pknA, or MBP-pknB cloned in MCS2 with or without sigA cloned in MCS1 were transformed in BL21 (DE3) codon plus cells. Lysates and His pulldowns were probed with anti-Thr(P), antiHis, and anti-MBP antibodies. *G*, quantification of three biological replicate data from above described *in vitro* transcription assay using phospho-SigA. *H*, *RvΔsigA* strain was electroporated with pNit-sigA, pNit-sigA_abl_, and pNit-sigA_Mim_ to generate *RvΔsigA:sigA*, *RvΔsigA::sigA*_*abl*_, and *RvΔsigA::sigA*_*Mim*_. WCLs were prepared from cultures inoculated at A_600_ of ∼0.05 and grown for 6 days in the 7H9 media containing 0.2 μM IVN in the presence or absence of 1 μg/ml ATc. 30 μg of WCLs from *Rv, RvΔsigA*, and *RvΔsigA*_*wt/mut*_ were resolved on 10% SDS-PAGE and probed with α-sigA and α-GroEL antibodies. *I*, *Rv, RvΔsigA*, and *RvΔSigA*_*wt/mut*_ cultures were seeded at A ∼0.05 in 7H9-ADC + 0.2 mM IVN, in the absence or presence of ATc, and CFUs were enumerated on day 6. The data represent mean CFU log_10_/ml ± SD of three independent replicates. *J*, murine peritoneal macrophage cells were infected with *Rv*, *RvΔsigA*, and *RvΔsigA*_*wt/mut*_ as described in Figure 3, and CFUs were enumerated at 96 h p.i. Data information: the data represent mean CFU log_10_/ml ± SD of three independent replicates. Statistical significance was analyzed using one-way ANOVA (Tukey test; GraphPad prism 9). ∗, *p* < 0.01; ∗∗, *p* < 0.001; ∗∗∗, *p* < 0.0001. ATc, anhydrotetracycline; CFU, colony forming unit; IVN, isovaleronitrile; SPR, surface plasmon resonance; WCL, whole-cell lysate.

Phosphorylation of ECF-σ factors by Serine/threonine kinases influences their interaction with anti-σ factors thereby regulating its function (34). High throughput phosphoproteomic studies identified phosphorylation of T69 and T71 residues in the unstructured N-terminal region of σ^A^ (29, 30). Depletion of PknA results in decreased phosphorylation of T69 and T71 residues in σ^A^ (29). In addition, S57 in σ^A^ was also identified to be a site for phosphorylation (unpublished data). We sought to examine the impact of σ^A^ phosphorylation on its interactions with the promoter region, *in vitro* transcription, and its function in *Mtb*. To evaluate the impact of phosphorylation, we generated σ^A^ phosphoablative mutants σ^A^_Abl_ (S_57_, T_69_, T_71_→A_57_, A_69_, A_71_) and phosphomimetic variants σ^A^_Mim_ (S_57_, T_69_, T_71_→D_57_, E_69_, E_71_) (Figs 7*A* and S5). These mutant factors *viz*., His-σ^A^, His-σ^A^_Abl_, & His-σ^A^_Mim_ purified from *E. coli* were used for interaction and *in vitro* transcription studies. Simultaneously, CD studies suggested the absence of gross changes in secondary structure among the σ^A^_Abl_ & σ^A^_Mim_ fragments (Fig. S6). SPR analysis suggested that the binding affinity of σ^A^_Abl_ mutant is ∼2 fold higher than σ^A^ (Fig. 7, *B* and *C*). The trend was similar to σ^A^_Mim_; however, the values were not statistically significant. Next, we performed *in vitro* transcription assay, as described earlier to evaluate the impact of σ^A^ phosphorylation on transcription. Compared with the WT σ^A^, both phosphoablative and mimetic mutants resulted in higher amounts of RNA product (Fig. 7, *D*–*E*). To further investigate the effect of SigA phosphorylation, we coexpressed His-SigA and MBP or MBP-PknA or MBP-PknB. We generated a single construct coexpressing both SigA and kinase with a Histidine and a MBP tag respectively. *E. coli* was used as a surrogate host to obtain phosphorylated SigA. It is apparent from the Western blot presented in Figure 7*F* that His-SigA, MBP, MBP-PknA, and MBP-PknB expressed robustly in the lysates (Fig. 7*F*). Subsequently, we validated the phosphorylation of SigA by PknA and PknB by probing the purified SigA with phosphor-threonine antibodies (Fig. 7*F*). We note that both PknA and PknB phosphorylate SigA efficiently *in vivo.* To evaluate the phosphorylation status of SigA, His-SigA was pulled down and probed with α-p-Thr and α-SigA antibodies. α-p-Thr blots indicate that both PknA and PknB phosphorylate SigA (Fig. 7*F*). In an *in vitro* transcription assay with phosphorylated SigA, we found that phosphorylated SigA (SigA-MBP-PknA/B) does not show significant change in transcript formation in comparison to unphosphorylated SigA (SigA-MBP) (Fig. 7*G*).

Subsequently, we subcloned these genes into the pNit-1 vector; the recombinant *Mtb* strains were evaluated for their ability to complement the depletion phenotype. The expression levels of σ^A^_wt/mut_ were found to be comparable with endogenous σ^A^ expression (Fig. 7*H*). While the WT almost wholly rescued the phenotype, both σ^A^_Abl_ and SigA_Mim_ partially rescued the growth defects *in vitro* and *ex vivo* (Fig. 7, I and *J*). The inability of σ^A^_Abl's_ to complement may be due to the possible interactions of the hydroxyl group with the initiator complex. On the other hand, the failure of σ^A^_Mim_ to completely complement may be due to the regulatory role of phosphorylation in the initiator complex. Together, these results suggest a partial role for phosphorylation in regulating the σ^A^-mediated transcription.

## Discussion

The response of *Mtb* to various host-derived stresses *in vivo* is coordinated by many regulators such as secretory virulence factors, one- and two-component signaling modules, serine/threonine protein kinases, and a multitude of transcription factors. Besides protecting *Mtb* from host immune response, these highly effective stress response strategies also contribute to antibiotic tolerance. Transcription factors, including σ factors, CarD, RbpA, WhiB family protein, and others, contribute to the regulation at the transcriptional level. σ factors are necessary for the recruitment of RNAP to the promoter. Thus, their levels in the cytosol impact the polymerase–promoter interaction and subsequent transcription (8, 9). While there is considerable literature on the functions of nonessential σ factors, insights into structure-function features and regulatory aspects of the essential σ factor σ^A^ *in vivo* remained elusive. This lacuna is in large part due to the nonavailability of genetic tools such as a conditional gene replacement mutant. This report sought to investigate many unanswered questions related to σ^A^ in *Mtb* by generating a tetracycline-regulatable conditional gene replacement mutant (Fig. 1).

Gene replacement mutants provide valuable information regarding the functional roles as well as the *in vitro*, *ex vivo*, and *in vivo* essentiality of a gene. High-throughput transposon mutagenesis studies suggested that except *sigA,* the remaining twelve σ factors are nonessential. Of these, except for *sigK,* gene replacement mutants were generated for the remaining eleven nonessential σ factors (28, 35, 36, 37, 38, 39, 40, 41, 42, 43, 44, 45). Even though these σ factors are nonessential for growth *in vitro*, deletion of *sigC, sigD, sigE, sigF, sigH,* and *sigL* leads to attenuated growth in murine or guinea pig models of infection (35, 36, 37, 38, 41, 43, 46, 47). *SigB* and *sigJ* are notable omissions, where their deletion had no impact on the growth *in vivo* (28, 42). Here, we created a conditional gene replacement mutant of *sigA* to determine its role. Results presented in Figure 1, Figure 2 show that *sigA* is essential for growth *in vitro*, *ex vivo*, and *in vivo*. This agrees with a previous report, wherein an anti-sense *sigA* RNA was used to downregulate its expression; the strain showed decreased growth in both macrophage and murine models (14). However, these experiments were performed for only 20 days in murine models. Importantly, conditional gene replacement generated in our study allowed us to evaluate the essentiality of *sigA* in an established infection (Fig. 2). Our results suggest that depletion of σ^A^ even from an established infection leads to mycobacterial death (Fig. 2). However, it has not escaped our attention that there were no further changes in the mycobacterial load between the sixth and 10th week despite continued depletion of *sigA*. We speculate that other stress-induced σ factors may partially compensate for the decreased levels of σ^A^ in chronic stages of infection in murine lungs. The same approach has been used previously to investigate the effect of GlmU depletion from fully infected lungs, wherein we observed complete clearance of pathogens (48). As compared to GlmU depletion, σ^A^ depletion from established infection produced a weaker phenotype. Based on this observation, we concluded that σ^A^ depletion may not be as crucial for pathogen survival during chronic infection.

Examining global transcriptional changes in the absence of transcription factors provides information on the subset of genes regulated by the factor. Depleting *sigA* for 4 days resulted in compromised RNA quality, and depletion for 2 days was insufficient (data not shown). Nevertheless, we compared analyzed RNA-sequ data from second depleted cells with third depleted cells. The large proportion of upregulation or downregulation DEGs, the difference became more pronounced when cells progressed from second to third depletion (data not shown). RNA-seq analysis of 3-day–depleted *RvΔsigA* samples showed differential expression of 2320 genes (Fig. 3). These include genes involved in critical metabolic pathways, nucleic acid biosynthesis, DNA replication, lipid, and protein synthesis. Energy metabolism generates ATP, which fuels cellular processes and thus represents one of the most critical pathways in an organism. In mycobacteria, 10 NADH dehydrogenases (*nuo A to J*) convert nicotinamide NADH to NAD^+^ (49), and results show that all 10 genes are downregulated upon σ^A^ depletion. Significantly, ∼30% of all the essential genes are differentially regulated upon σ^A^ depletion, which is likely to be the reason for the cell death. We also observed upregulation of σ^B^ in σ^A^-depleted samples both at RNA and protein levels (Fig. 3), suggesting that mycobacteria may be compensating for the absence of σ^A^ by upregulating the expression of σ^B^.

Overexpression of σ^B^ upregulates transcription of PE-PGRS proteins, culture filtrate antigens, ribosomal proteins, the keto-acyl synthase, KasA, and the regulatory proteins WhiB2 and IdeR (50). However, the expression of other σ factor genes is unaltered, suggesting that σ^B^ may serve as a terminal modulator of the σ factor regulatory cascade (50). Chip Seq experiments revealed that in *M. smegmatis*, 72 out of 200 promoters detected are shared between both σ^A^ and σ^B^ (45). The expression levels of σ^B^ at both RNA and protein levels are higher upon the depletion of σ^A^ (Fig. 3). Despite having considerable overlap at amino acid levels with σ^A^ (∼64%), σ^B^ failed to rescue the *sigA* depletion phenotype (Fig. 4). In a previous study, RNA-seq analysis performed with *M. smegmatis* σ^B^ mutant showed 125 DEGs with 100 genes upregulated and 25 genes downregulated (27). We find that in *Mtb*, 433 genes are DEGs upon σ^B^ deletion. The differences in the number of DEGs between the two studies are likely due to (a) differences in the strains used for the studies and (b) differences in the cut-offs applied. Among the 433 DEGs obtained upon deletion of *Mtb* σ^B^, 283 genes overlapped with σ^A^ DEGs. Among the 1168 downregulation genes σ^A^ regulon, only 28 genes seem to be dependent on higher expression of σ^B^ (Fig. 5). The striking difference between these σ factors is the absence of the N-terminal region in σ^B^. We made attempts to generate a chimera wherein the N-terminal domain of σ^A^ was fused to σ^B^. However, we could not detect the expression of the chimeric protein suggesting that fusion protein may be unstable (data not shown).

Orthologs of σ^A^ possesses varying lengths of N-terminal extension. These range from 83 aa in *Bacillus subtilis*, 95 in *E. coli*, and 163 in *M. smegmatis* (Fig. S7). However, the N-terminal polypeptide stretch observed in σ^A^ is significantly longer at 221 aa. Domain σ_1.1_ (σR.1.1) is involved in abrogating nonproductive interactions of free σ^A^ with the promoter (51, 52). Upon the formation of holoenzyme, domain σ_1.1._ occupies RNAP active site channel as a DNA mimic (6, 53). In σ^A^, the 1.1 region is not well defined. Results suggest that deletion of amino acids between 132-179 abrogates σ^A^ functionality (Fig. 6). We detected high levels of negatively charged amino acid residues in regions beyond 132. Based on the data, we speculate that the amino acids beyond 132 may be playing a role akin to σ_1.1_.

Serine/threonine-protein kinase–mediated phosphorylation regulates many cellular processes in mycobacteria, including transcription. An example of immediate relevance is the ECF σ factors σ^H^ and its cognate anti-σ factor RshA. Both are targets of PknB, and the phosphorylation of RshA interferes with σ^H^–RshA interactions (54, 55). PknD-mediated phosphorylation of Rv0516c inhibits its interaction with the anti–anti-σ factor and eventually influences the genes regulated by σ^F^ (56). In the case of the primary σ factor σ^A^, high throughput phosphoproteomic studies led to the identification of residues T69 and T71 as sites for phosphorylation. Both sites lie in the intrinsically disordered N-terminal stretch in σ^A^. Subsequently, we identified an additional phosphorylation on S57 in a high throughput phosphoproteomic analysis performed recently (unpublished data). Does phosphorylation alter promoter recognition or transcription initiation? This was clearly not the case as absence of phosphorylation or σ^A^ mutants mimicking phosphorylation had no impact on either DNA binding or *in vitro* transcription (Fig. 4). Intriguingly, neither mutant could completely restore the function of σ^A^ *in vitro* growth nor *ex vivo* (Fig. 4). Despite phosphorylation playing a partial role in *ex vivo* survival, we did not observe any significant differences in *in vitro* transcription initiation when we used the phosphorylated form of σ^A^ (Fig. 7*H*). According to these results, the phosphorylation of σ^A^ may modulate the nature and strength of σ^A^ interactions with other proteins, thus regulating promoter binding.

Together, these studies suggest that the long N-terminal intrinsically disordered polypeptide stretch in σ^A^ performs a distinct functional and regulatory role in this primary σ factor. The primary-like σ^B^ lacks this stretch and cannot functionally replace σ^A^. The distinctive regulons of these two σ factors also highlight the functional differences. These observations, alongside the finding that depleting *sigA* in an established *Mtb* infection leads to mycobacterial death, rationalizes the critical role of this essential initiation factor in the survival and pathogenicity of *Mtb*. There are certain sigma factor deletion mutant strains that exhibit attenuated disease progression and prolonged survival in immunocompetent hosts in spite of no growth deficits under *in vivo* conditions. They may serve as good vaccine candidates if they persist in the host at a high level of infection and stimulate the immune response without inflicting detrimental pathological changes. A number of mycobacterial strains exhibit this kind of phenotype, including *sigF, sigH, sigE, sigD*, and *sigC* mutants (35, 36, 37, 38, 41, 43, 46, 47). Nevertheless, we have not evaluated the immune response after depleting σ^A^. Further experiments are needed to determine the therapeutic potential of σ^A^.

## Experimental procedures

### Animal experimentation

Animal experiment protocols were reviewed and approved by the Institutional Animal Ethics Committee of the National Institute of Immunology, New Delhi, India (the approval number is IAEC# 462/18). The experiments were carried out as per the guidelines issued by the Committee for the Purpose of Control and Supervision of Experiments on Animals (CPCSEA), Govt. of India.

### Bacterial strains and culturing

The list of bacterial strains used is given in Table 1. Cultures of *Mtb* (H37Rv or mutant strains) strains were grown in Middlebrook 7H9 medium (BD Biosciences) supplemented with 10% albumin, dextrose, catalase, NaCl, and 0.2% glycerol (Sigma), or 7H11 agar (BD Biosciences) 10% OADC (oleic acid added to ADC). Log phase cultures grown in the absence of ATc were used for seeding new cultures at A ∼0.05. The expression of the episomal copy of SigA (from pNit constructs) was induced by adding 0.2 μM isovaleronitrile (IVN). Cultures were grown in the absence or presence of 1 mg/ml ATc, and the growth was monitored by enumerating CFUs every day or 6 days postinoculation depending on the experiment. All the experiments were performed in biological triplicates.

**Table 1:** Strains used in the study

Strains	Description	Source
*Rv*	WT *Mycobacterium tuberculosis* H37Rv (*Rv*) strain	ATCC
*Rv::pNit*	*Rv* strain electroporated with episomal pNit-1 vector; Kan^r^	This study
*Rv::pN-sigA*	*Rv* strain electroporated with pNit-*sigA* construct	This study
*Rv::pN-sigA*	*Rv* electroporated with pNit-*sigA* construct	This study
*RvΔsigA*	*sigA conditional mutant. sigA* gene expression is under the regulation of tetracycline inducible promoter.	This study
*RvΔsigA::pN*	*RvΔsigA* electroporated with pNit-1 vector	This study
*RvΔsigA::sigA*	*RvΔsigA* electroporated with pNit-*sigA* construct	This study
*RvΔsigA::sigA*_*Δ42*_	*RvΔsigA* electroporated with pNit-*sigA*_*Δ42*_ construct	This study
*RvΔsigA::sigA*_*Δ92*_	*RvΔsigA* electroporated with pNit-*sigA*_*Δ92*_ construct	This study
*RvΔsigA::sigA*_*Δ132*_	*RvΔsigA* electroporated with pNit-*sigA*_*Δ132*_ construct	This study
*RvΔsigA::sigA*_*Δ179*_	*RvΔsigA* electroporated with pNit-*sigA*_*Δ179*_ construct	This study
*RvΔsigA::sigA*_*Abl*_	*RvΔsigA* electroporated with pNit-*sigAA*_*bl*_ construct	This study
*RvΔsigA::sigA*_*Mim*_	*RvΔsigA* electroporated with pNit-*sigA*_*Mim*_ construct	This study
*RvΔsigA::sig*_*pro*_*-luc*	*RvΔsigA* electroporated with pSWN-*sig*_*pro*_*-luc* construct	This study
*Rv::sigB*	*Rv* electroporated with pNit-*sigB* construct	This study
*RvΔsigA::sigB*	*RvΔsigA* electroporated with pNit-*sigB* construct	This study
*BL21(DE3)*	WT *Escherichia coli* strain	This study

### Expression and purification of recombinant *M. tuberculosis* protein in *E. coli* system

RbpA and CarD from *Mtb* were cloned into pET-28a vector, and the proteins were overexpressed in *E. coli* BL21(DE3) cells. The recombinant proteins were purified by Ni-affinity chromatography in 50 mM Tris–HCl pH8.0, 500 mM NaCl, 5% glycerol, and eluted using 20 to 500 mM gradient of imidazole. The eluted fractions were resolved on 15% SDS-PAGE and then concentrated using ultrafiltration (Merck). The proteins were buffer exchanged to 20 mM Tris–HCl pH 8, 150 mM NaCl, 5% glycerol. Subunits of *Mtb* RNAP were expressed and purified from *E. coli* BL21(DE3) cotransformed with pETBC and pACYCZA (encoding *rpoB, rpoC, rpoZ*, and *rpoA* subunits; a kind gift from Banerjee *et al.*, 2014) (57). Core RNAP was purified as described previously, and final fractions containing RNAP were pooled, concentrated to nearly 1 mg/ml, and stored as aliquots at −80 °C. *sigA* was cloned into MCS-I of MBP/MBP-PknA/MBP-PknB-pDuet vector (58). SigA was coexpressed with MBP, MBP-PknA, or MBPPknB, respectively, in *E. coli* surrogate host as we described previously (58).

### Generation of mutant strain

*SigA* or *sigB* was PCR amplified from *Mtb H37Rv* genomic DNA with phosphorylated forward and reverse harboring *NdeI* and *HindIII* sites. Amplicons were purified and cloned into the *SmaI* digested and dephosphorylated pUC19 vector. The *sigA* insert was released with *NdeI-HindIII* digestion and cloned into corresponding sites in *Mycobacterial-E. coli* shuttle plasmid pFICTO (59). pFICTO-sigA was electroporated into *Rv* to generate merodiploid strain *Rv::sigA*, which expresses FLAG-SigA (3X-FLAG) from the L5 loci and *sigA* from the native loci. Upon the addition of ATc, the expression of FLAG-SigA shuts down as the tet repressor binds with its cognate operator sequences. To generate Allelic exchange substrate (AES), ∼1 kb left-hand– and right-hand–flanking sequences of *sigA* were amplified. Amplicons were digested with PflMI and ligated with oriE+ λ cos (1.6 kb) and *hygr-sacB* (3.6 kb) cassettes obtained after digesting pYUB1474 (32). AES was linearized with *PacI* and cloned into the corresponding site in the temperature-sensitive phAE159 shuttle phagemid. The gene replacement mutant *RvΔsigA* was generated with the help of specialized transduction (32) by transducing *Rv::sigA* merodiploid strain with the temperature-sensitive phagemid. Genomic DNA was prepared from the recombinant colonies, and recombination at the native sigA loci was confirmed by performing multiple PCRs. AES for making *RvΔsigB* mutant was generated as described above, harboring 1 kb upstream and downstream flanks. The mutant was generated with the help of the recombineering method (60), as described previously. The recombination at the native *sigB* loci in the resulting colonies was confirmed with the help of multiple PCRs.

### Generation of complementation constructs

Site-directed mutants in σ^A^ were generated with the help of overlapping PCRs. *sigA* or *sigA*_*Mu*t_ were subcloned into *NdeI-HindIII* sites in the pNit-1 (ref) vector to generate pNit-SigA or pNit-SigA_Mut_ constructs. The N-terminal deletion constructs of σ^A^ were generated by PCR amplifying, then using different N-terminal forward primers harboring the *NdeI* site and the reverse primer harboring the *HindIII* site. They were cloned into a pNit-1 vector to generate pNit-SigA_del_ constructs. Similarly, *the sigB* gene was cloned into the pNit-3F vector (61) to generate the pNit-SigB construct. The presence of mutations at the desired location was confirmed by sequencing. *RvΔsigA* was electroporated with pNit-SigA or pNit-SigA_Mut_, or pNit-SigA_del_, or pNit-SigB constructs to generate *RvΔsigA::sigA or RvΔsigA::sigA*_Mut_ or *RvΔsigA::sigA*_del_ or *RvΔsigA::sigB* strains, respectively. Various complementation strains generated in the study are described in Table 2. The expression of the episomal copy of SigA or SigA_Mut_ or SigA_Del_ or SigB was induced by adding 0.2 μM IVN.

**Table 2:** Oligos used in the study

Ref No	Description	Sequence (5′ – 3′)
STL-324	*sigA* gene forward with NdeI site	CACCCAT ATGGCAGCGACCAAAGCAAG
STL-325	*sigA* gene reverse with HindIII site	AGCTAAG CTTAGTCCAGGTAGTCGCGCAG
STL-320	*sigA*-AES 5′ flank forward- DraIII & HpaI site	CACCTTTTCACAAA GTG GTTAACCGACACAGCGTCATGGCTT
STL-321	*sigA*-AES 5′ flank reverse with DraIII site	TTTTTTTTCACTTCGTGGCCCGCTTCGCGGGTGGGGAGC
STL-322	*sigA*-AES 3′ flank forward harboring DraIII	CACCTTTTCACAGAGTG GGCCTTACCGACGGCCAGCCG
STL-323	*sigA*-AES 3′ flank reverse with DraIII & HpaI	TTTTTTTTCACCTTGTGGTTAACGGCTCCGGAGGGCAGCTTGGC
STL-330	*sigB* gene forward harboring NdeI	CACCCATATGGCCGATGCACCCACAAG
STL-331	*sigB* gene forward harboring HindIII	AGCTAAGCTTAGCTGGCGTACGACCGCAG
STL-326	*sigB*-AES 5′ flank forward with PflMI & SnaBI	CACCTTTTCCATAAATTGGTACGTATACCCATGACCGCGCTCTCAG
STL-327	*sigB*-AES 5′ flank reverse harboring PflMI	TTTTTTTTCCATTTCTTGGCTTGGCCAGTTCGACTTCACC
STL-328	*sigB*-AES 3′ flank forward harboring PflMI	TTTTTTTTCCATCTTTTGGTACGTAGACATCGCGGTCACCGGCTACC
STL-339	*sigB*-AES 3′ flank reverse with PflMI & SnaBI	TTTTTTTT CCATCTTTTGGTACGTAGACATCGCGGTCACCGGCTACC
STL-737	*sigA* forward qRT primer	CGCGACATGATGTGGATCTG
STL-738	*sigA* reverse qRT primer	CCCCTTGGTGTAGTCGAACT
STL-752	*sigB* gene reverse harboring HindIII	CACCAAGCTTATGGCAGCGACCAAAGCA
BSL-291	*Δ*42 *sigA* forward harboring NdeI	CACCCATATGGGCTCCCCACCCGCGAAG
BSL-292	*Δ*92 *sigA* forward harboring NdeI	CACCCATATGGGCCACGCGACCAAGCCA
BSL-293	*Δ*132 *sigA* forward harboring NdeI	CACCCATATGGGCGAGGACCTCGACGTT
BSL-294	*Δ*179 *sigA* forward harboring NdeI	CACCCATATGGGCCAGACCGCCGATGAC
STL-1030	*sigA* (S75 to A75) forward	AAGCCCGCGGCCCGG**GCA**GTCAAGCCCGCCTCG
STL-1031	*sigA* (S75 to A75) reverse	CGAGGCGGGCTTGACTGCCCGGGCCGCGGGCTT
STL-1032	*sigA* (S75 to D75) forward	GACACTACGACCAGC**GCA**ATCCCGAAAAGGAAG
STL-1033	*sigA* (S75 to D75) reverse	CTTCCTTTTCGGGAT**TGC**GCTGGTCGTAGTGTC
BSL-638	*sigA* (T69 & T71 to A69 & A71) forward	CCCCAGGACACTACG**GCA**AGC**GCA**ATCCCGAAA
BSL-639	*sigA* (T69 & T71 to A69 & A71) reverse	TTTCGGGATTGCGCTTGCCGTAGTGTCCTGGGG
BSL-640	*sigA* (T69 & T71 to E69 & E71) forward	CCCCAGGACACTACG**GCA**AGC**GCA**ATCCCGAAA
BSL-641	*sigA* (T69 & T71 to E69 & E71) reverse	TTTCGGGATTGCGCTTGCCGTAGTGTCCTGGGG
BSL-698	*sigA-sigB* fusion forward	GGTGATTTCGTCTGGGATGCACCCACAAGGGCCA
BSL-699	*sigA-sigB* fusion reverse	TGGCCCTTGTGGGTGCATCCCAGACGAAATCACC
BSL-821	*nuoA* forward qRT primer	ACAGTAACCGCCCTGTATGA
BSL-822	*nuoA* reverse qRT primer	CCCTGCCCACAGCCCTTCCG
BSL-823	*nuoB* forward qRT primer	AGACCCATTTGGGATGTTGG
BSL-824	*nuoB* reverse qRT primer	GCCCGTGGCTACTAAAGAAC
BSL-825	glmU forward qRT primer	TCGGGCTGGCAGGTCGCCGG
BSL-826	glmU reverse qRT primer	CGTCGGCGACAATCGGCGCC
BSL-827	lpqD forward qRT primer	GCTACACGGGTCGGGGCATG
BSL-828	lpqD reverse qRT primer	ACCAGGTGTACGGGCAGGCG
BSL-829	ctaE forward qRT primer	AGCGCCGGTAACTCCGATCA
BSL-830	ctaE reverse qRT primer	GGGCGTGGCCGGGGTTGCCG
BSL-831	*whiB* forward qRT primer	AAGTCGTCCTCCCTGGCAG
BSL-832	*whiB* reverse qRT primer	GCAGCCGCTGGGCGACCCGC
BSL-833	*PE22* forward qRT primer	GCGATCGAGATGATGGCGAC
BSL-834	*PE22* reverse qRT primer	CACACCCATGGCCAGAACCC
BSL-835	*icl1* forward qRT primer	GCTGGACGCCGACACGCTGG
BSL-836	*icl1* reverse qRT primer	GATTTCCCGCTGCGATGGTG
BSL-837	*sigB* forward qRT primer	CCGACATGCCCGGTTCCGGC
BSL-838	*sigB* reverse qRT primer	GGCCCGGAAGAATCTCGACT
BSL-839	*espK* forward qRT primer	CCGACATGCCCGGTTCCGGC
BSL-840	*espK* reverse qRT primer	AGAACGAAGAACAGGCCCAT

Bold and underlined text indicate substitution mutations at this positions.

### Lysate preparation, western blots, and growth kinetics

*Rv and RvsigA::sigA*_*wt/mut/del*_ strains grown in the absence of ATc or IVN were seeded at A ∼0.05 in the presence of 0.2 μM IVN and absence or presence of 1 μg/ml ATc. *In vitro* growth in the 7H9-ADC medium was performed for either 6 days or as indicated in the figure/legend. The survival was evaluated by enumerating CFUs on 7H11-OADC-agar plates. Whole-cell lysates (WCLs) were prepared as described previously (62). WCLs were estimated using the Bradford protein estimation method, and 30 μg WCLs were resolved on 10% SDS-PAGE, transferred to a nitrocellulose membrane, and probed with α-SigA, α-GroEL1 antibodies generated previously in the lab (62).

### *Ex vivo* peritoneal macrophages infections

BALB/c mice were injected with 4% thioglycolate solution (Difco) in the peritoneal cavity, and 96 h post-injection, peritoneal macrophage was isolated and seeded in RPMI media containing 10% fetal bovine serum. Log phase cultures of Mtb strains passed through a 26 gauge needle to create single-cell suspensions. Peritoneal macrophages were infected with *Rv* or *RvΔsigA* or *RvΔsigA::sigA* or *RvΔsigA::sigA*_Mut_ or *RvΔsigA::sigA*_del_ or *RvΔsigA::sigRv,* at MOI of 1:5 (cell: bacteria). Four hours p.i, media was removed and cells were washed thrice to remove extracellular bacteria. The cells were replenished with RPMI media containing 10% fetal bovine serum with or without 0.2 μM IVN or 1 μg/ml ATc. 24, 48, 72, or 96 h p.i, cells were lysed in 100 μl of 0.05% SDS, and CFUs were enumerated different dilutions on OADC-containing 7H11 agar plates.

### *In vivo* mice infections

To investigate the physiological impact of *sigA* depletion on bacterial survival in the host, we performed murine infection experiments as described previously (62). Dox hydrochloride (1 mg/kg with 5% dextrose in drinking water) was provided to *Rv* and *RvΔsigA*-infected mice as indicated in legends/figures, either from the time of the infection (day 1) or after infection establishment (14 days p.i). CFUs were enumerated at the indicated times.

### Luciferase assays

Five hundred basepair sequence just upstream of *sigA* ORF was cloned into an integrative shuttle plasmid (pSW-luc_SApr_) at the *ScaI-NdeI* site. The luciferase (*luc*) ORF was cloned into *NdeI-HindIII* sites. *Rv* and *RvΔsigA* strains were electroporated with pSWN-sigA_pro_-luc to generate *Rv::sig*_*pro-luc*_ and *RvΔsigA::sig*_*pro-luc*_ strains. WCL was prepared as described above, and Luciferase assays were performed as described previously (62).

### SPR studies

Interaction of σA, its N-terminal truncations, and its phosphoablative and phosphomimetic mutations with *rrnAP3* promoter were performed in Biacore 2000 instrument (Biacore,). The sense and anti-sense strand of *rrnAP3* promoter DNA (−70 to +20) was PCR amplified from *Mtb* genome. The sense strand was synthesized with 5′ biotinylation modification (Sigma Aldrich). The strands were annealed before the immobilization by mixing the sense: anti-sense strand at a molecular ratio of 1:2 in Sodium Saline citrate buffer. Biotinylated *rrnAP3* (5′) promoter fragment was immobilized on an SA chip at a surface density of 985 RU ng/mm^2^. The experiment was executed in running buffer comprising 25 mM Hepes (pH 8), 250 mM NaCl, and 5% glycerol. The study used various concentrations of purified His-σ^A^ and His-σ^A^_Mut_ as analytes. The first channel of SA chip was not immobilized with any biotinylated promoter and was used as a control for the binding studies. BIA-evaluation software program was used to evaluate interaction kinetics. The σ^A^ and *rrnAP3* promoter interaction sensograms were used as control for calculating both σ^A^ N-terminal truncation constructs (Fig. 6*B*) and σ^A^ phosphomutants promoter–binding kinetics (Fig. 7, *B* and *C*).

### *In vitro* transcription assay

Transcription was carried out in 5 μl transcription buffer (TB, 10 mM Tris–HCl pH 7.9, 70 mM K-glutamate, 0.1 mM EDTA, 1 mM DTT, 5% glycerol, 5 mM MgCl2, and 2 mM MnCl2). The promoter *rrnAP3* (−100 to +150) was PCR amplified from *Mtb* genomic DNA, and the amplicons were purified. RNAP holoenzyme was assembled by mixing 100 nM core RNAP with 500 nM σ subunit followed by 15 min incubation at 37 °C. Subsequently, 2 μM RbpA and 4 μM of CarD were added to the reaction and incubated for 10 min. The above mixture was incubated with a 40 nM rrnAP3 promoter fragment at 37 °C for 5 min in the presence of 1 μl heparin (0.5 mg/ml) to inhibit the nonspecific RNA–DNA complex. RNA synthesis was initiated by addition of 1 μl NTP mix (final concentration: 0.1 mM of ATP, GTP, CTP, 10 μM of UTP, and 0.5 μCi α[^32^P] UTP) and the reaction was carried out for 45 min at 37 °C. RNA products were purified by precipitating using 1/10th volume of 3 M sodium acetate (pH 5.2), glycogen (1 μg/μl), and 2.5 volumes of 100% cold ethanol. The samples were centrifuged at 10,000 rpm, the pellet was washed with 70% cold ethanol, and the pellet was eventually resuspended in 20 μl nuclease-free water. 2× RNA loading dye (80% formamide, 10 mM EDTA, 0.01% Bromophenol Blue, 0.01% Xylene Cyanol) was added to the samples, heated at 95 °C for 5 min chilled on ice, and resolved on 5% Urea-PAGE. Transcription products were visualized using Phosphorimager and quantified by using Quantity One software (Biorad).

### RNA isolation and qRT-PCRs

Total RNA was isolated from exponentially growing bacteria cells. To isolate total RNA, cells were inoculated at 0.05 A in the presence or absence of ATc and allowed to grow for 3 days. Cells were resuspended in TRIzol reagent (Invitrogen), and total RNA was isolated from following mycobacterial cells and performed qRT-PCRs as described perviously (62).

### Transcriptomics study

Agilent 2100 Bioanalyser (Agilent RNA 6000 Nano Kit) or 4200 Tape station system was used to determine RNA integrity. RNA-seq was performed using samples with RIN values higher than 7. Sequencing was performed at the Centre for cellular and molecular biology core sequencing facility. Illumina adapters and low-quality reads were eliminated from raw sequencing reads using cutadapt. Low read quality scores (<20) and <36 bp were discarded. The processed reads were then mapped to the *Mtb* H37RV, downloaded from https://ftp.ncbi.nlm.nih.gov/genomes/refseq/bacteria/Mycobacterium_tuberculosis/reference/GCF_000195955.2_ASM19595v2/, using hisat2 with default parameters. Uniquely aligned reads were counted using feature Counts of the Subread package. There were 4008 genes in the gtf file, for which we had the count information. The gtf was modified to remove 200 bp from either end of SigA/SigB. Genes with a total read count 10 across all the samples were removed, resulting in 3987 genes for the SigA knockdown experiment and 3999 genes for the SigB KO experiment. DESeq2 was used to analyze differential gene expression. A gene was considered to be differentially expressed if the adjusted *p*-value was less than 0.05 and the absolute log_2_ fold change was greater than 0.5. Raw read counts were normalized with rlog, provided by DESeq2 package for PCA plot and heat map.

### Functional enrichment analysis

For functional enrichment analysis, DAVID web services were used (https://david.ncifcrf.gov/). We only used the GO terms and KEGG pathways for enrichment analysis. We only plotted the top 10 enriched GO terms/KEGG pathways based on gene counts.

### Statistical analysis

One-way ANOVA was used to determine the significance of the data. The data sets were plotted and statistical significance was calculated with GraphPad Prism version 9 followed by minimal modifications using Adobe Illustrator (2021). The corresponding author can provide the source data for this study upon request.

## Data availability

RNAseq data are available at the NCBI Gene Expression Omnibus Database, accession number. GEO (GSE197742). (https://www.ncbi.nlm.nih.gov/geo/query/acc.cgi?acc=GSE197742).

## Supporting information

This article contains supporting information.

## Conflict of interest

B. S. is a Senior Research Fellow. The authors declare that they have no conflicts of interest with the contents of this article.
