# Supplementary material for: The unique N-terminal region of Mycobacterium tuberculosis sigma factor A plays a dominant role in the essential function of this protein
Source: J Biol Chem. 2023 Jan 20;299(3):102933. doi: 10.1016/j.jbc.2023.102933 (PMC10011835; doi:10.1016/j.jbc.2023.102933)
Supplement: Supplementary Figures [file mmc6.pdf]

Figure S1

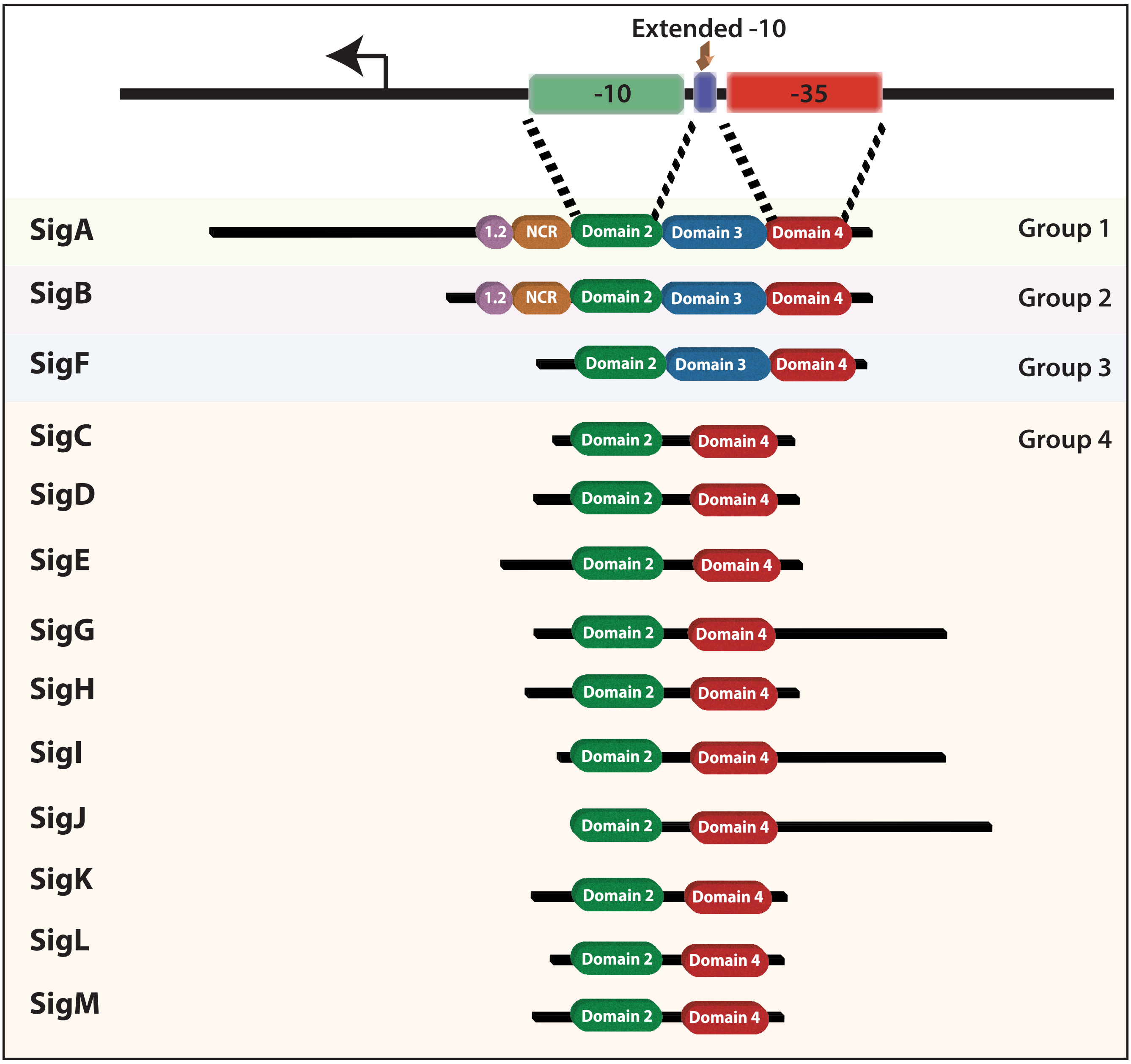

Extended -10
-10
-35
SigA
1.2
NCR
Domain 2
Domain 3
Domain 4
Group 1
SigB
1.2
NCR
Domain 2
Domain 3
Domain 4
Group 2
SigF
Domain 2
Domain 3
Domain 4
Group 3
SigC
Domain 2
Domain 4
Group 4
SigD
Domain 2
Domain 4
SigE
Domain 2
Domain 4
SigG
Domain 2
Domain 4
SigH
Domain 2
Domain 4
SigI
Domain 2
Domain 4
SigJ
Domain 2
Domain 4
SigK
Domain 2
Domain 4
SigL
Domain 2
Domain 4
SigM
Domain 2
Domain 4

Figure S2

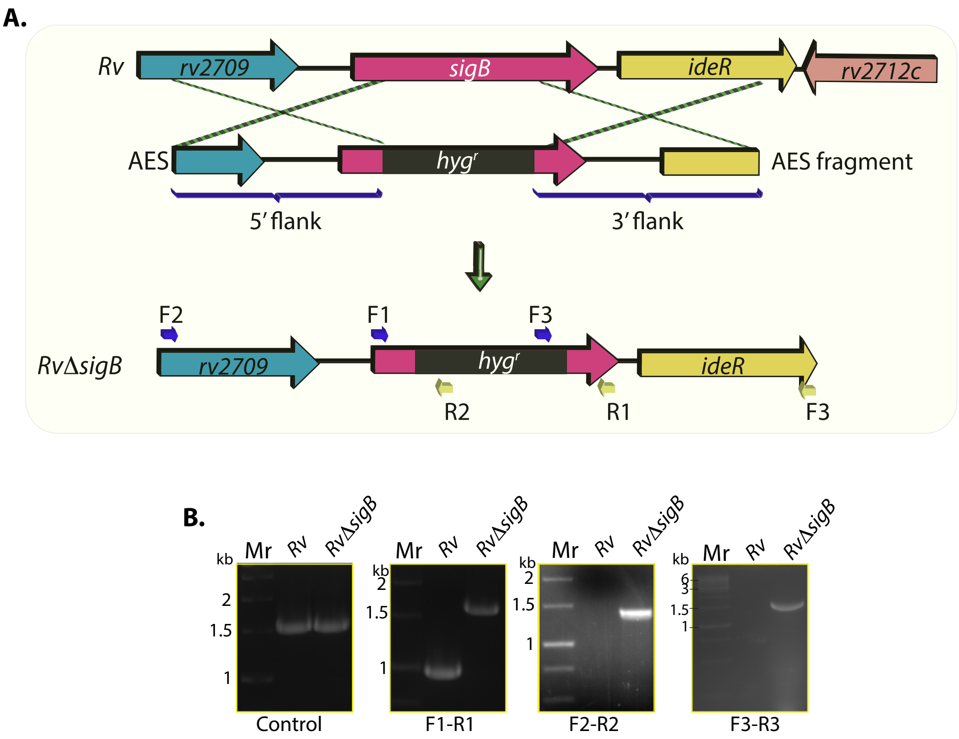

A.
Rv
rv2709
sigB
ideR
rv2712c
AES
hygr
AES fragment
5′ flank
3′ flank
F2
F1
F3
RvΔsigB
rv2709
hygr
ideR
R2
R1
F3
B.
kb
Mr
Rv
RvΔsigB
2
1.5
1
Control
F1-R1
F2-R2
F3-R3
6
3

Figure S3

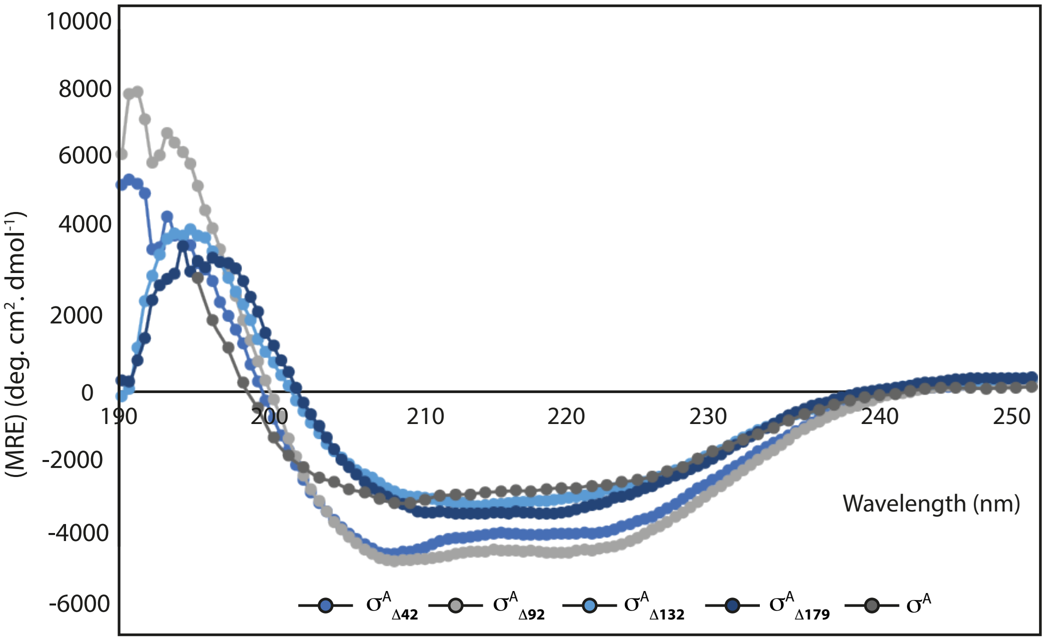
10000
8000
6000
4000
2000
0
-2000
-4000
-6000
(MRE) (deg. cm². dmol⁻¹)
190
200
210
220
230
240
250
Wavelength (nm)
σA Δ42
σA Δ92
σA Δ132
σA Δ179
σA

Figure S4

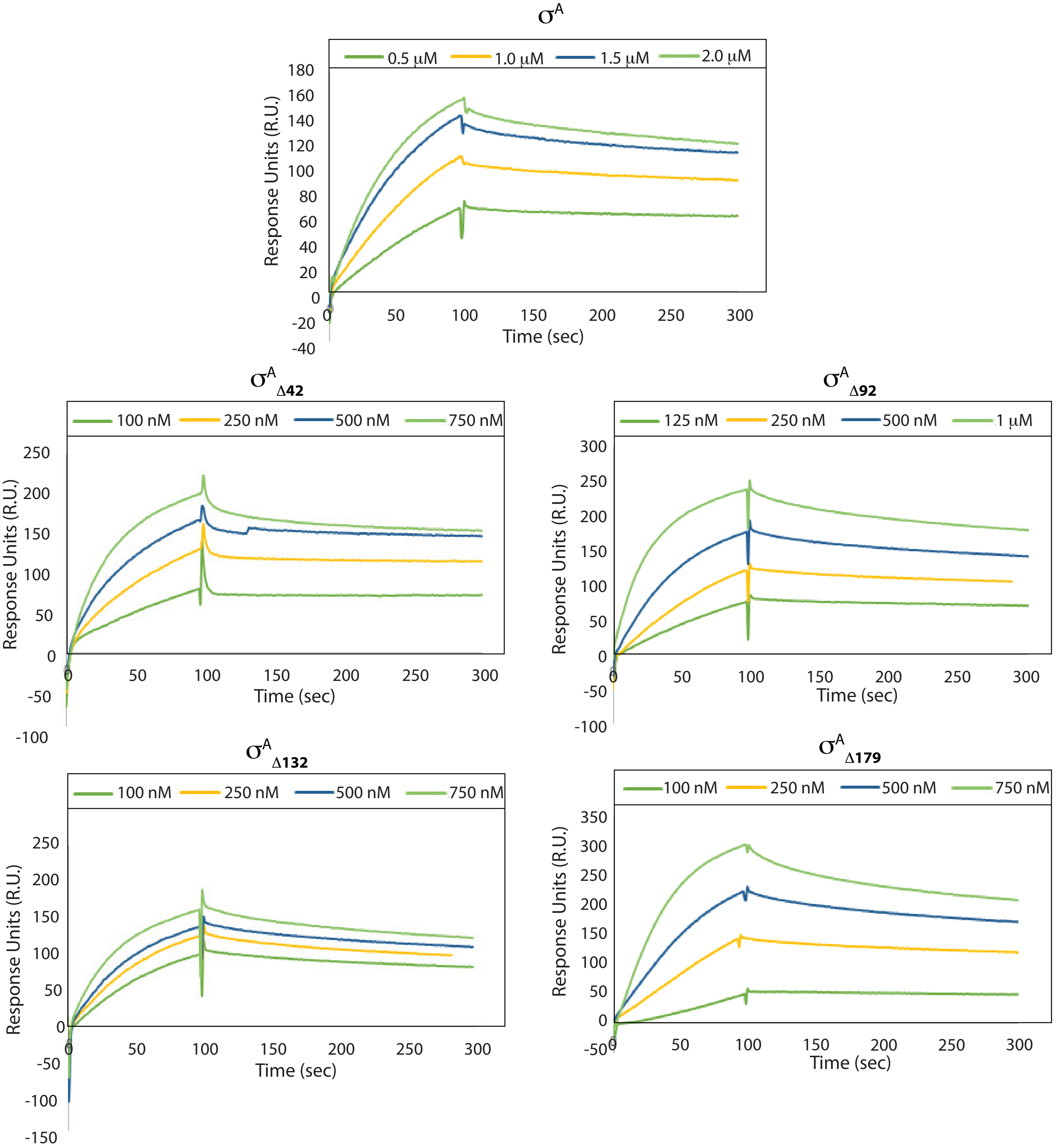
σA
0.5 μM
1.0 μM
1.5 μM
2.0 μM
Response Units (R.U.)
180
160
140
120
100
80
60
40
20
0
-20
-40
0
50
100
150
200
250
300
Time (sec)
σA Δ42
100 nM
250 nM
500 nM
750 nM
Response Units (R.U.)
250
200
150
100
50
0
-50
-100
Time (sec)
σA Δ92
125 nM
250 nM
500 nM
1 μM
Response Units (R.U.)
300
Time (sec)
σA Δ132
100 nM
250 nM
500 nM
750 nM
Response Units (R.U.)
-150
Time (sec)
σA Δ179
100 nM
250 nM
500 nM
750 nM
Response Units (R.U.)
350
Time (sec)

Figure S5

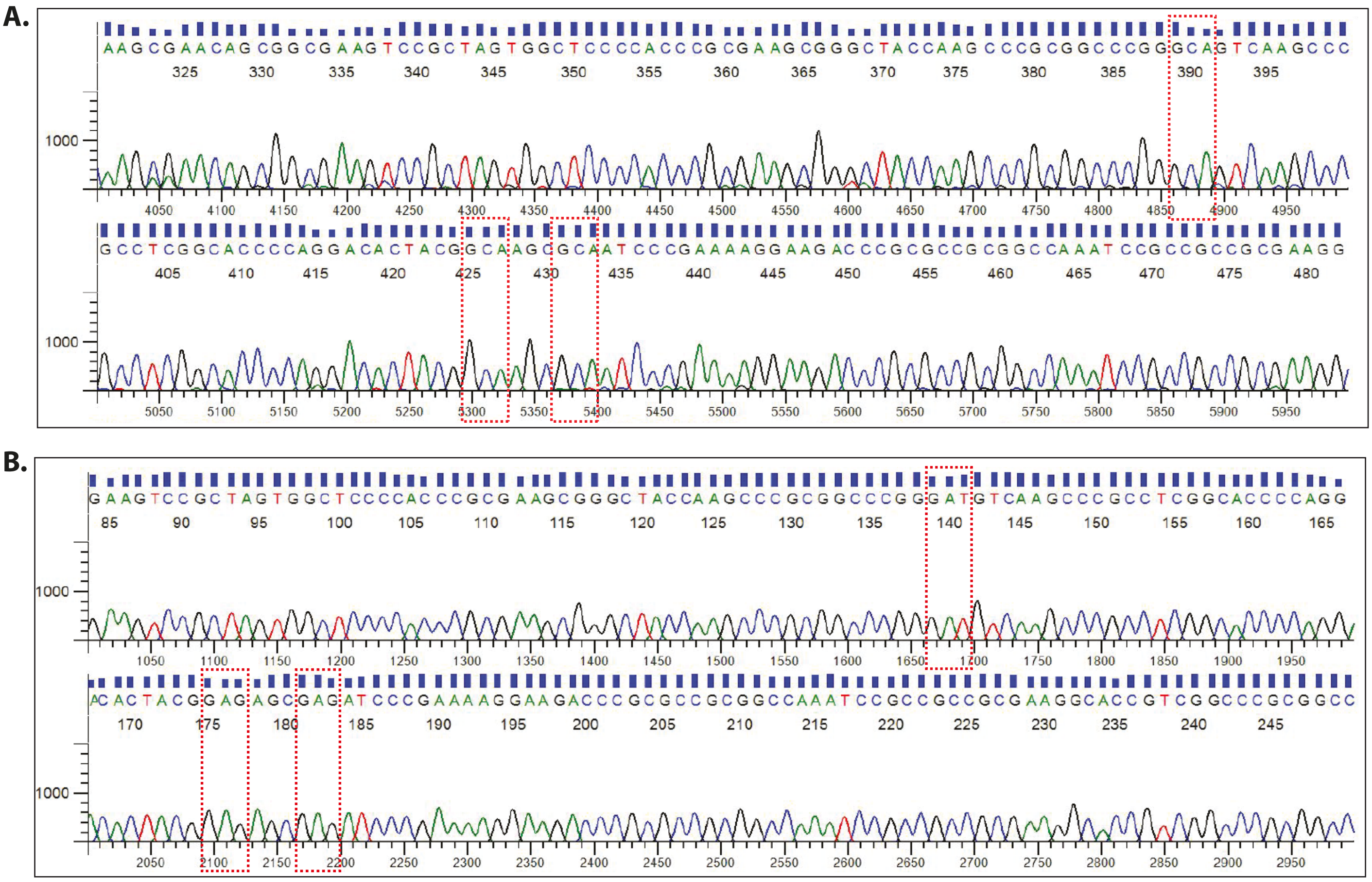

Figure S6

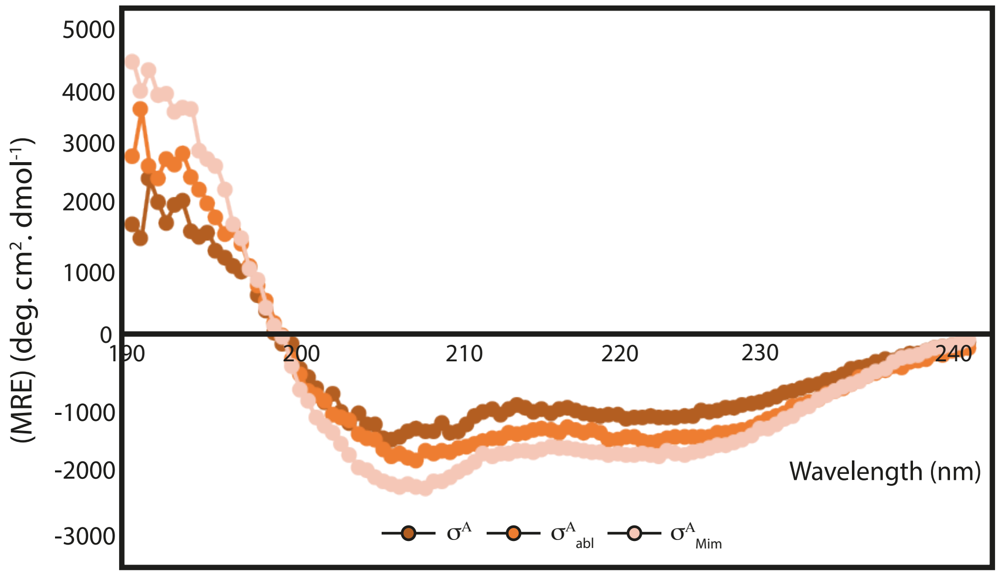

5000
4000
3000
2000
1000
0
-1000
-2000
-3000
(MRE) (deg. cm². dmol⁻¹)
190
200
210
220
230
240
Wavelength (nm)
σA
σAabl
σAMim

Figure S7

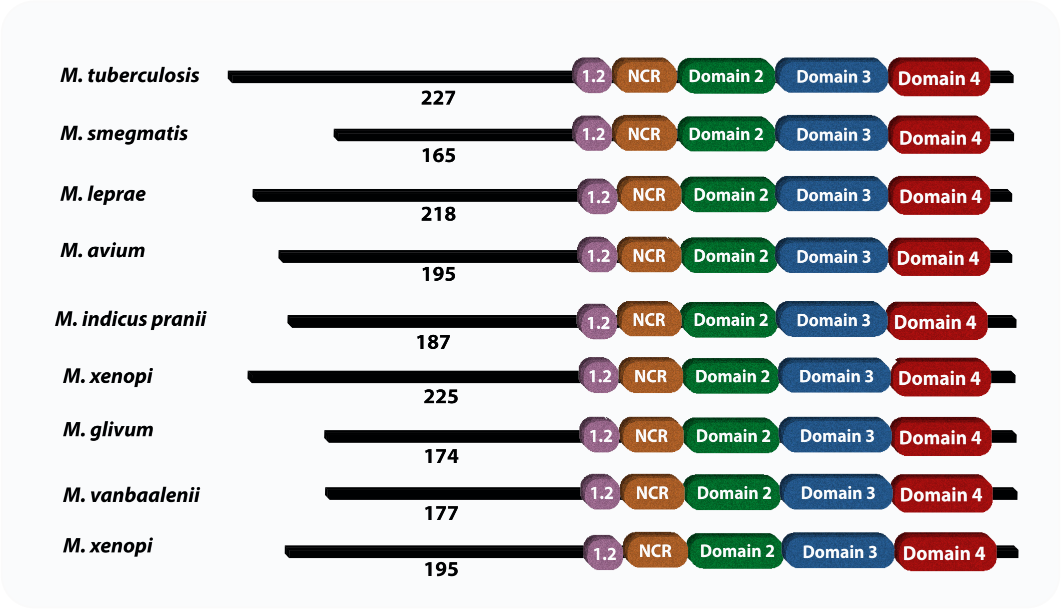
M. tuberculosis
1.2
NCR
Domain 2
Domain 3
Domain 4
227
M. smegmatis
165
M. leprae
218
M. avium
195
M. indicus pranii
187
M. xenopi
225
M. glivum
174
M. vanbaalenii
177
M. xenopi
195
